# Mosquito‐repellent controlled‐release formulations for fighting infectious diseases

**DOI:** 10.1186/s12936-021-03681-7

**Published:** 2021-03-24

**Authors:** António B. Mapossa, Walter W. Focke, Robert K. Tewo, René Androsch, Taneshka Kruger

**Affiliations:** 1 Department of Chemical Engineering, Institute of Applied Materials , University of Pretoria, Lynnwood Road, Pretoria, South Africa; 2 UP Institute for Sustainable Malaria Control & MRC Collaborating Centre for Malaria Research, School of Health Systems and Public Health, University of Pretoria, Private Bag X20, Hatfield, 0028 Pretoria, South Africa; 3 Department of Chemical Engineering, Vaal University of Technology, Private Bag X021, 1911 Vanderbijlpark, South Africa; 4 Interdisciplinary Center for Transfer-oriented Research in Natural Sciences, Martin Luther University Halle-Wittenberg, 06099 Halle/Saale, Germany

**Keywords:** Malaria, Vector control, Mosquito repellent, controlled‐release formulations, Kinetic model

## Abstract

Malaria is a principal cause of illness and death in countries where the disease is endemic. Personal protection against mosquitoes using repellents could be a useful method that can reduce and/or prevent transmission of mosquito-borne diseases. The available repellent products, such as creams, roll-ons, and sprays for personal protection against mosquitoes, lack adequate long-term efficacy. In most cases, they need to be re-applied or replaced frequently. The encapsulation and release of the repellents from several matrices has risen as an alternative process for the development of invention of repellent based systems. The present work reviews various studies about the development and use of repellent controlled-release formulations such as polymer microcapsules, polymer microporous formulations, polymer micelles, nanoemulsions, solid-lipid nanoparticles, liposomes and cyclodextrins as new tools for mosquito-borne malaria control in the outdoor environment. Furthermore, investigation on the mathematical modelling used for the release rate of repellents is discussed in depth by exploring the Higuchi, Korsmeyer-Peppas, Weibull models, as well as the recently developed Mapossa model. Therefore, the studies searched suggest that the final repellents based-product should not only be effective against mosquito vectors of malaria parasites, but also reduce the biting frequency of other mosquitoes transmitting diseases, such as dengue fever, chikungunya, yellow fever and Zika virus. In this way, they will contribute to the improvement in overall public health and social well-being.

**Supplementary Information:**

The online version contains supplementary material available at 10.1186/s12936-021-03681-7.

## Background

Mosquitoes are vectors of numerous diseases, including malaria, chikungunya, Zika virus, or yellow fever. Among them, malaria is a principal cause of illness and death in countries where the disease is endemic. According to the World Health Organization (WHO) [1], in 2018, around 228 million malaria cases were reported with an estimated number of 405,000 fatalities [2]. Most of the reported cases occurred in sub-Saharan Africa, with children younger than five years and pregnant women considered most prone to malaria. During that year, the WHO reported that around 67 % (272,000) out of the total number of deaths were the deaths of children [2]. The United Nations’ Sustainable Development Goal 3 (health and wellbeing for all), specifically target 3.3, contains the bold commitment to end the epidemic of malaria by the year 2030. This may be achieved by preventing or reducing the incidence of infective mosquito bites that can also cause secondary infections, pain, discomfort and allergic reactions in sensitive individuals and systemic reactions, such as urticaria and angioedema of the skin [3–5].

Over the years, malaria control has been increasingly aimed at eliminating or reducing mosquito populations. Several methods are available for controlling the malaria vectors. Among them, long-lasting insecticidal nets (LLINs) and indoor residual spraying (IRS) are the most important control strategies recommended by the WHO. However, these methods are not effective in an outdoor environment, where people spend time during the day and early evening.

Braack et al. [6] reported on the biting behaviour of African malaria vectors to identify where they tend to bite on the human body. The vectors used in the study were *Anopheles arabiensis* from Malahlapanga in South Africa, and *Anopheles gambiae* and *Anopheles funestus* from northern Uganda. The results showed that more than 93 % of mosquito bites occur on the ankles and feet of people seated or standing outdoors. Additionally, the study reported that mosquitoes are attracted to the smell of the feet and ankles. However, if the feet and ankles are protected or covered, the mosquitoes will not bite above the ankle but seek alternative hosts with non-covered ankles and feet. Additionally, Reddy et al. [7] studied the behaviour of *An. gambiae* and *Anopheles melas* outdoors on Bioko Island, Equatorial Guinea. The study showed that high levels of outdoor biting by mosquitoes occurred at night and during the early evening and morning. These findings highlight the need for further studies about the importance and urgency of developing new methods to control mosquito-borne diseases when humans are outdoors.

Personal protection against mosquitoes by the application of repellents has become a useful practice that can reduce and/or prevent transmission of many insect-borne diseases. Mosquito repellents are known as volatile chemicals which, when applied on human skin, repel mosquitoes in the opposite direction from its source, thus discouraging contact and bites [8]. Numerous repellent-based products, such as creams, roll-ons and sprays, are available on the market for outdoor protection. However, most of these applications have a very short period of protection of a few hours only [9]. This also includes topical skin applications, requiring frequent application due to environmental effects such as excessive sweating, humidity and insect activity. Due to the use of repellent products requiring frequent application, their use would not be affordable to poorer communities. Longer periods of protection from mosquito bites are thus required.

Research activities to obtain long-lasting repellency include a study by Izadi et al. [10], who evaluated the performance of the repellent ethyl butylacetylaminopropionate (IR3535^®^) blended with nonanoic acid against the biting of *An. arabiensis*, lasting for up to four hours. The blend also caused the mortality of the mosquitoes used in the study. N’Guessan et al. [11] reported an excellent activity of repellency and mortality of nets treated with repellent N,N-diethyl-3-methylbenzamide (DEET) against *Aedes aegypti* for 6 months. Akhtar [12] developed a natural mosquito repellent-based polymer matrix and evaluated the repellent release from the polymer matrix. Sibanda et al. [13] reported on the slow release of DEET from polymer fibres. Their results provided effectiveness against *An. arabiensis* for up to 20 weeks. In addition, Mapossa et al. [14] investigated repellents, such as DEET and Icaridin, incoporated in microporous polyolefins strands against *An. arabiensis*. It was found that the strands with 20 % and 30 % of repellents provided a good repellency activity against *An. arabiensis* for up to twelve weeks. The results obtained suggest the development of an alternative tool such as repellent-based products (example bracelets/anklets containing repellent) for long protection duration against mosquitoes. According to these studies, polymer systems hold the most promise for the controlled release of repellents. A polymer is a substance made up of macromolecules composed of many repeating subunits.

In this paper, a review of formulations for controlled-release of repellents is provided in terms of: (i) basic principles of preparation of the repellent-based formulations; (ii) mechanism of repellent release from formulations, and (iii) the effectiveness against mosquitoes. Furthermore, mathematical models for release of repellents and their use are also presented and discussed.

## Wearable device’s based on mosquitoes repellents available on the markert

Previous studies have showed an association between the application of personal protection products and a reduction in mosquito bites and disease incidence [15]. For example, Schreck and Kline [16] reported that the repellents-treated military clothing demonstrated to be effective in significantly reducing mosquito bites in the covered regions. Repellent such as DEET-based on soaps has been demonstrated to successfully reduce malaria infections [17, 18]. Furthermore, the efficacy of different spray-on repellents on various species of mosquitoes has been reported in several studies [19].

Additionally, a study conducted by Rodriguez et al. [20, 21] evaluated the efficacy of various commercial controlled release devices based on repellents against *Aedes aegypti*. The results demonstrated that from five wearable devices evaluated such as: OFF!^®^ Clip-On, PIC^®^ Personal Sonic Mosquito Repeller, Mosquitavert^®^ Repellent Bracelet, Mosquito-No!^™^ Repellent Bracelet, and InvisabandTM); one candle (Cutter^®^ Citro Guard), and five sprays (Cutter^®^ Lemon Eucalyptus, All Terrain^®^ Kids Herbal Armor^™^, Avon^®^ Skin-So-Soft Bug Guard Plus Picaridin, Repel^®^ Sportsmen Max Formula^®^, and Ben’s^®^ Tick & Insect Repellent) the only wearable device that fared well in the study was OFF!^®^ Clip-On, which features a nebulizer to evaporate its repellent chemical, Metofluthrin. The PIC^®^ Personal Sonic Mosquito Repeller and bracelets showed no significant reduction in mosquito attraction.

The five spray-based on repellents evaluated exhibited significant, although varying, levels of reduction in mosquito attraction in the test. Cutter^®^ Lemon Eucalyptus (30 wt% of *p*-menthane-3,8-diol derived from lemon scented eucalyptus leaves, known by its chemical acronym, PMD) and Ben’s^®^ Tick & Insect Repellent (98 wt-% DEET) were the most effective [20]. This finding confirms the findings of numerous other studies that found DEET and PMD the most effective and longest lasting mosquito repellents currently available.

Finally, the results of the study demonstrated that not all commercially available mosquito repellents are effective in repelling mosquitoes and that efficacy is also dependent on the species of mosquito that is repelled. Overall, the results from the study confirmed that DEET-based products are the most effective mosquito repellents on the market [20, 21]. Therefore, the researchers also observed that their study focused on the efficacy of wearable devices and spray-on repellents against *Ae. aegypti* females and that further studies are required to explore the efficacy of these interventions on repelling other mosquito species. Additionally, more studies about the efficacy of the wearable device’s based on mosquito repellents available on the markert are summarized in Table 1.

**Table 1:** Previous studies about the efficacy of mosquito repellents, repellent-based controlled release devices and repellent-based products available on the market against mosquitoes

Controlled-release devices	Polymeric material	Repellent	Preparation method	Reference
*Microporous polymer*				
Microporous polymer	HDPE/EVA	DEET	TIPS method by Electrospining	[13]
Microporous polymer	LLDPE and EVA/clay nanocomposite	DEET and Icaridin	Thermally Induced Phase Separation method	[14]
Microporous polymer	LLDPE	Citronellal	Thermally Induced Phase Separation method	[117]
Microporous polymer	PLA	DEET	Thermally Induced Phase Separation method	[123]
Microporous polymer	Cellulose acetate or polyvinylpyrrolidone micro/nanofibrous matrices	Citronella (*Cymbopogon nardus*) oil	Electrospining	[177]
Microporous polymer	Bio-degradable polymer (PLA/PBAT)	Pine (*Pinus sylvestris*) essential oil	Thermally Induced Phase Separation method	[178]
*Nanoemulsions/microemulsion*				
Nanoemulsion	Montanov®82 (a mixture of cetearyl alcohol and cocoyl glucoside)	Citronella (*C. nardus*) oil	High-pressure homogenization	[56]
Nanoemulsion	Montanov®82 (a mixture of cetearyl alcohol and cocoyl glucoside)	Citronella (*C. nardus*) oil, hairy basil (*Ocimum americanum*) oil and vetiver (*Vetiveria zizanioides*) oil	High-pressure homogenization	[55]
Nanoemulsion	Tween 20 [polyoxyethylene (20) sorbitan monolaurate]	Neem seed (*Azadirachta indica*) oil	Ultrasonication	[50]
Nanoemulsion	Sorbitane trioleate and polyoxyethylene (20) oleyl ether with mean HLB number 1.8 and 15.0	_D_-limonene	Ultrasonication	[179]
Nanoemulsion	Tween 80 (HLB = 15) and SPAN 80 (HLB = 4.3)	Citronella (*C. nardus*) oil	Ultrasonication	[57]
Nanoemulsion	Polyethylene glycol sorbitan monooleate (Tween® 80) and sorbitan monooleate (Span® 80)	Clove (*Syzygium aromaticum*) oil	Ultrasonication	[180]
Nanoemulsion	EL-20, EL-40, EL-60, and EL-80 [polyoxyethylene (20, 40, 60, and 80)	D-limonene	Phase transition composition	[58]
Nanoemulsion	Poloxamer 407	Eugenol and thymol	High-energy stirring	[61]
Polymeric microcapsules	Gelatin and Gum arabic	Citronella (*C. nardus*) oil	Complex coacervation	[31]
Polymeric microcapsules	Copolymer poly(maleic anhydride-st-methyl vinyl ether − MAMVE)	Jojoba (*Simmondsia chinensis*) oil	Interfacial polycondensation	[181]
Polymeric microcapsules	Gelatin and ethyl cellulose	*Zanthoxylum limonella* oil	Emulsion solvent evaporation	[182]
Polymeric microcapsules	Polysaccharide	DEET	Interfacial precipitation	[37]
Polymeric microcapsules	Poly(methyl methacrylate) (PMMA)	DEET	Interfacial polymerization	[36]
Polymeric microcapsules	Gelatin and Gum arabic	Citronella (*C. nardus*) oil	Complex coacervation	[32]
Polymeric microcapsules	Polyurethane	Citronella (*C. nardus*) oil	Interfacial polymerization	[30]
Polymeric microcapsules	Polyvinyl alcohol (PVA), gum arabic (GA) and whey protein isolate/maltodextrin (WPI/MD)	Neem seed (*A. indica*) oil	Spray drying	[28]
Polymeric microcapsules	Acacia gum	Citronella (*C. nardus*) oil	Spray drying	[183]
Polymeric microcapsules	Gelatin	Citronella (*C. nardus*) oil	Simple coacervation	[33]
Polymeric microcapsules	Polyurethane	DEET	Interfacial polycondensation	[44]
Polymeric microcapsules	Polyester	Citronella (*C. nardus*) oil	Complex coacervation	[43]
Polymeric nano/microcapsules	Ethyl cellulose shell	Limonene	Simple coacervation	[34]
Polymeric microcapsules	Cetyl alcohol core/PEG 3350 and carboxymethylcellulose wall	DEET and Essential (*Alpinia galangal, Citrus grandis and Citrus aurantifolia*) oil	Interfacial polymerization	[184]
Polymeric microcapsules	Polyurea and Polyurethane	DEET	Interfacial polymerization	[185]
Polymeric microcapsules	Polyurea (PU) and poly (methyl methacrylate) (PMMA)	DEET	Interfacial polymerization and Solvent evaporation	[186]
Polymeric microcapsules	Polysaccharides	DEET	Interfacial precipitation	[38]
Polymeric microcapsules	Carboxymethylated Tamarind Gum	Citronella (*C. nardus*) oil	Spray drying	[29]
Solid Lipid Nanoparticles				
Solid Lipid Nanoparticles	Compritol 888 ATO as lipid and Poloxamer 188	Essential Oil	High-pressure homogenization	[71]
Solid Lipid Nanoparticles	Tween^®^ 20	DEET	Melt-dispersion	[70]
Solid Lipid Nanoparticles	Polyethylene glycol (PEG)	Garlic (*Allium sativum*) essential oil	Melt-dispersion	[72]
Solid Lipid Nanoparticles	Tween® 80 (polysorbate 80, polyoxyethylene sorbitan monooleate)	Geranium (*Pelargonium graveolens*) essential oil	Ultrasonic-solvent emulsification	[68]
Solid Lipid Nanoparticles	Tween® 80 (polysorbate 80, polyoxyethylene sorbitan monooleate)	Geranium (*P. graveolens*) essential oil	Ultrasonic-solvent emulsification	[66]
*Cyclodextrins*				
Cyclodextrin	β-cyclodextrin	*Citrus sinensis* essential oil (CSEO)	Paste complexation and Co-precipitation	[83]
Cyclodextrin	β-cyclodextrin	Citronella (*C. nardus*) oil, Citronellal and Citronellol	Kneading	[86]
Cyclodextrin	γ-cyclodextrin	DEET	Paste complexation	[187]
Cyclodextrin	β-cyclodextrin	Thyme (*Thymus vulgaris*) oil	Mixing and heating,	[188]
Cyclodextrin	β-cyclodextrin	Limonene	Conventional impregnation and coating	[80]
Cyclodextrin	β-cyclodextrin	Geraniol and Linalool	Physical mixture, Slurry complexation and Paste complexation	[189]
Cyclodextrin	β-cyclodextrin	Carvacrol and Linalool	Kneading	[190]
Cyclodextrin	β-cyclodextrin	Citronella (*C. nardus*) oil	Mixing and heating	[88]
Polymeric micelles	Poloxamer 407 (Pluronic^®^ F127)^a^	Essential oil components (EOCs)	High-energy stirring	[79]
Polymeric micelles	Poly(ethylene glycol) (PEG)	Diethylphenylacetamide (DEPA)	Polymerization followed by Phase Inversion Temperature (PIT) emulsification method	[78]
Polymeric micelles	Poloxamer 407 (Pluronic^®^ F127)	DEET	High-speed Homogenizer	[77]
Polymeric micelles	Poloxamer 407 (Pluronic^®^ F127)	IR3535	High-speed Homogenizer	[191]

*The repellents-based on controlled release formulations were prepared in the laboratory and evaluated against different species of mosquitoes. Therefore, the manufacture names were provided only for the repellents-products or wearable devices commercially available on the market.

## Mosquito‐repellent controlled‐release formulations

Controlled release is a technology, which is used to retain the supply of the reagent and to permit the release of the active ingredient to the target at a controlled rate, in an ideal case maintaining its concentration in the formulation within the optimal limits over a prolonged or required period of time [22–24]. The advantages of this technology include: (i) activity prolongation by providing continuously low amounts of a repellent at a level sufficient to perform its function over a long period of time; (ii) environmental pollution reduction, and (iii) cost reduction by eliminating the time and cost of repeated and over-applications [24]. This reduces the undesirable side-effects of compound losses such as that of repellents by evaporation and degradation, or masking of any odour, since toxic material becomes chemically non-toxic when combined with polymers [23–25].

In order to select the best system to release a sufficient amount of repellent and to reach the desired effect with minimum biological or ecological adverse risks, the following characteristics need to be considered: (i) the nature of the polymer (degree of cross-linking, thermal behaviour, compatibility with the active agent); (ii) the stability of the polymer/repellent combination during processing; (iii) the desired release rate; (iv) shape and size of the final product; (v) protection time; (vi) seasonal conditions, and (vii) cost and ease of formulation and application [23].

**Fig. 1:**
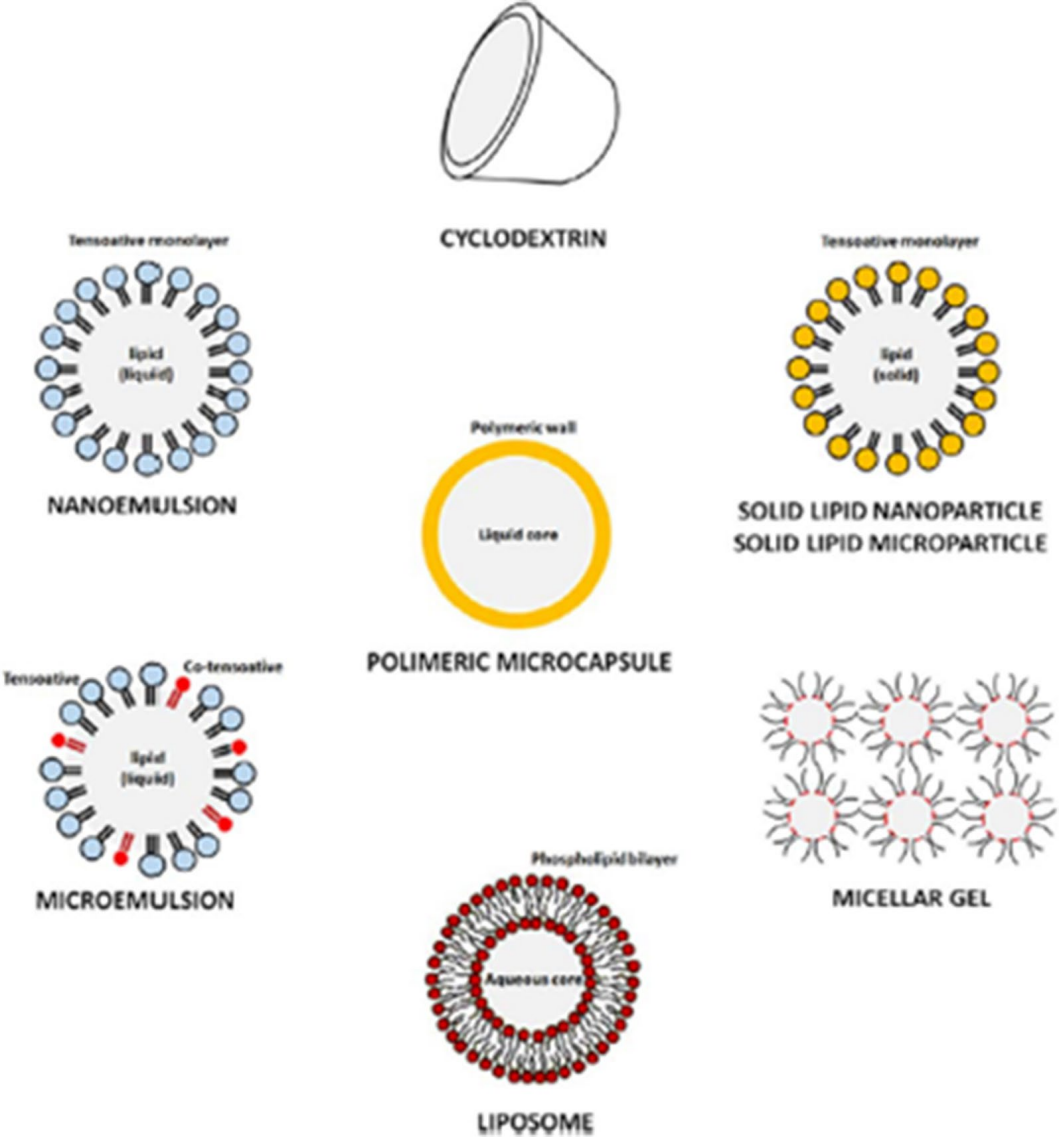
Different systems of controlled-repellent-release [35]. Republished with permission from Elsevier

Different systems of controlled-release are presented in Fig. 1, including polymeric microcapsules, nanoemulsions, cyclodextrins, solid lipid nanoparticles, liposomes and polymer-based micellar or microporous systems. Table 2 summarizes/lists previous studies of repellent-based controlled-release formulations obtained by several technologies including the types of polymers and repellents used to fabricate those systems.

**Table 2:** Controlled-release systems of mosquito repellent actives, polymeric material, mosquito repellent, and their preparation method

Mosquitoes	Product, active ingredient and concentration	Protection (%)	Repellency time (h)	Manufacturer	References
*Culex quinquefasciatus*	Citriodiol® (30 %) based repellent (Mosiguard®)	100	3	Mosi-guard	[184]
*C. quinquefasciatus*	Citronella KAPS®	> 84	4	KAPS Mosquito Repellent Patch 12 s	[184]
*C. quinquefasciatus*	Citronella MozAway®	> 84	4	MozAway	[184]
*C. quinquefasciatus*	Citronella BioZ Natural®	> 81	4	BioZ Natural	[184]
*Aedes aegypti*	Citronellal	> 71	1	*	[192]
*A. aegypti*	Citronellol	> 77	1	*	[192]
*A. aegypti*	Geraniol	78	1	*	[192]
*C. quinquefasciatus*	Microencapsulated formulation of Essential oil (*Citrus aurantifolia*) 20 %	> 85	6	*	[184]
*C. quinquefasciatus*	Microencapsulated of Essential oil (*C. aurantifolia*) 15 %	> 84	6	*	[184]
*C. quinquefasciatus*	Microencapsulated of Essential oil (*C. aurantifolia*) 10 %	> 75	6	*	[184]
*C. quinquefasciatus*	Microencapsulated of Essential oil (*C. aurantifolia*) 5 %	> 63	6	*	[184]
*C. quinquefasciatus*	Non-encapsulated of Essential oil (*C. aurantifolia* 20 %	> 71	6	*	[184]
*C. quinquefasciatus*	Essential oil (*Citrus grandis*) 20 % microencapsuled	> 86	6	*	[184]
*C. quinquefasciatus*	Essential oil (*C.s grandis*) 15 % microencapsuled	> 83	6	*	[184]
*C. quinquefasciatus*	Essential oil (*C. grandis*) 10 % micrencapsulated	> 74	6	*	[184]
*C. quinquefasciatus*	Essential oil (*C. grandis*) 5 % micrencapsulated	> 65	6	*	[184]
*C. quinquefasciatus*	Non-encapsulated of Essential oil (*C. grandis*) 20 %	> 72	6	*	[184]
*C. quinquefasciatus*	Microencapsulated of Essential oil (*Alpinias galanga*) 20 %	> 88	6	*	[184]
*C. quinquefasciatus*	Microencapsulated of Essential oil (*A.s galanga*) 15 %	> 83	6	*	[184]
*C. quinquefasciatus*	Microencapsulated of Essential oil (*A. galanga*) 10 %	> 76	6	*	[184]
*C. quinquefasciatus*	Microencapsulated of Essential oil (*A. galanga*) 5 %	> 71	6	*	[184]
*C. quinquefasciatus*	Non-encapsulated of Essential oil (*A. galanga*) 20 %	> 73	6	*	[184]
*Anopheles subpictus*	Essential oil (*Zingiber officinale Rosc.* 5 mg/m^2^*)*	> 85	3	*	[193]
*An. subpictus*	Essential oil (*Rosmarinus officinalis L*.5 mg/m^2^)	> 68	3	*	[193]
*An. subpictus*	Essential oil (*Cymbopogan citrates Stapf*. 5 mg/m^2^)	> 74	3	*	[193]
*An. subpictus*	Essential oil from (*Cinnamomum zeylanicum L.* 5 mg/m^2^*)*	> 61	3	*	[193]
*Anopheles darlingi*	30 % PMD in ethanol	97	4	*	[194]
*Aedes ochlerotatus taeniorhynchus*	15 % PMD (derived by acid modification of Citronellal)	99	5	*	[194]
*A. aegypti*	*Hazomalania voyronii* fresh bark essential oil (EO) 100 %	> 82	0.5	*	[195]
*C. quinquefasciatus*	*Boesenbergia rotunda oil* 10 %	-	4	*	[196]
*C. quinquefasciatus*	*Curcuma zedoaria oil* 10 %	-	3	*	[196]
*C. quinquefasciatus*	*Zingiber cassumunar oil* 10 %	-	2	*	[196]
*C. quinquefasciatus*	*L. petiolata oil* 10 %	-	3	*	[196]
*A. aegypti*	*Hazomalania voyronii* fresh bark essential oil (EO) 50 %	> 78	0.5	*	[195]
*C. quinquefasciatus*	*H. voyronii* fresh bark essential oil (EO) 100 %	> 98	0.5	*	[195]
*Aedes albopictus*	Citronella oil 5 %	> 57	2	*	[197]
*A. aegypti*	Citrodiol into the ethylcellulose nanofibrous	100	816	*	[198]
*Culex tritaeniorhynchus*	Essential oil (*Z. officinale Rosc.* 5 mg/m^2^*)*	> 88	3	*	[193]
*C. tritaeniorhynchus*	Essential oil (*R. officinalis L*.5 mg/m^2^)	> 71	3	*	[193]
*C. tritaeniorhynchus*	Essential oil (*C. citrates Stapf*. 5 mg/m^2^)	> 79	3	*	[193]
*C. tritaeniorhynchus*	Essential oil from (*C. zeylanicum L.* 5 mg/m^2^*)*	> 64	3	*	[193]
*An. darlingi*	15 % DEET in Ethanol	85	4	*	[194]
*A. ochlerotatus taeniorhynchus*	15 % DEET in Ethanol	92	5	*	[194]
*C. quinquefasciatus*	20 % DEET	-	4	*	[196]
*A. aegypti*	20 % DEET	-	4	*	[196]
*Anopheles gambiae and C. quinquefasciatus*	DEET-treated nets (DEET-TN)	-	1008	*	[199]
*A. albopictus*	Skinsations® Spray-DEET 7 %	-	5	Spectrum Division of United Industries Corporation	[200]
*A. albopictus*	Off! Spray DEET 15 %	-	> 7	S.C. Johnson & Son Inc.	[200]
*A. aegypti*	DEET 20 %	> 82	5	*	[201]
*Aedes communis*	DEET (The amount was not specified)	98	4	*	[202]
*A. communis*	DEET (The amount was not specified)	74	6	*	[202]
*A. communis*	DEET (The amount was not specified)	56	8	*	[202]
*A. communis*	DEET + AI3-37220 (The amount was not specified)	98	4	*	[202]
*A. communis*	DEET + AI3-37220 (The amount was not specified)	95	6	*	[202]
*A. communis*	DEET + AI3-37220 (The amount was not specified)	76	8	*	[202]
*A. aegypti*	OFF! Deep Woods-DEET 23.8 %	-	> 5	S.C. Johnson & Son Inc.	[203]
*A. aegypti*	Sawyer Controlled Release®-DEET 20 %	-	> 3	Sawyer	[203]
*A. aegypti*	OFF! Skintastic-DEET 6.65 %	-	> 1	S.C. Johnson & Son Inc.	[203]
*A. aegypti*	OFF! Skintastic for Kids-DEET 4.75 %	-	> 1	S.C. Johnson & Son Inc.	[203]
*A. aegypti*	DEET 25 %	100	6	*	[204]
*A. aegypti*	DEET 25 % + Vanillin 5 %	100	6	*	[204]
*A. aegypti*	DEET 20 % in ethanol	100	7	*	[204]
*A. aegypti*	DEET 20 % in ethanol	100	8	*	[205]
*Aedes vigilax*	DEET 34.6 % Army repellent personal	> 95	5	*	[206]
*A. albopictus*	DEET 10 %	100	4	*	[207]
*A. albopictus*	DEET 10 %	> 88	6	*	[207]
*A. albopictus*	DEET 10 %	> 77	8	*	[207]
*A. aegypti*	DEET 12 % Cream	> 96	> 6	*	[208]
*Anopheles* spp.	DEET 20 %	> 88	4	*	[206]
*Anopheles spp.*	DEET 20 %	> 74	5	*	[206]
*An. gambiae*	DEET 30 %	> 88	7	*	[209]
*Anopheles stephensi*	DEET 12 % Cream	100	11	*	[208]
*Anopheles culicifacies*	DEET 12 % Cream	100	11	*	[208]
*Anopheles annularis*	DEET 12 % Cream	100	11	*	[208]
*An. subpictus*	DEET 12 % Cream	100	11	*	[208]
*A. albopictus*	Insectan Spray DEET 24 %	> 90	6	*	[210]
*Anopheles arabiensis*	Socks – DEET 20 %	> 90	3360	*	[13]
*C. quinquefasciatus*	Microencapsulated DEET 20 %	98	6	*	[184]
*C. quinquefasciatus*	Microencapsulated DEET 15 %	95	6	*	[184]
*C. quinquefasciatus*	Microencapsulated DEET 10 %	85	6	*	[184]
*C. quinquefasciatus*	Microencapsulated DEET 5 %	83	6	*	[184]
*C. quinquefasciatus*	Non-Encapsulated DEET 20 %	91	6	*	[184]
*C. quinquefasciatus*	DEET 1 %	90	-	*	[211]
*A. aegypti*	DEET 1 %	77	-	*	[211]
*A. aegypti*	OFF Family – DEET < 10 %	-	2	S.C. Johnson & Son Inc.	[9]
*A. aegypti*	Repelex – DEET < 10 %	-	2	US CHEMCO Supply & Service	[9]
*A. aegypti*	Mosquitoff – DEET 10 %	-	> 2	S.C. Johnson & Son Inc.	[9]
*A. aegypti*	SBP – Icaridin 15 %	-	5	Sawyer Products	[9]
*A. aegypti*	OFF kids – DEET < 10 %	-	2	S.C. Johnson & Son Inc.	[9]
*A. aegypti*	Muriel – DEET < 10 %	-	2		[9]
*A. aegypti*	Kor Yor 15® DEET	-	> 7	S.C. Johnson & Son Inc.	[212]
*C. quinquefasciatus*	Kor Yor 15® DEET	-	> 7	S.C. Johnson & Son Inc.	[212]
*An. arabiensis*	LLDPE strands-DEET 20 %	79	2016	*	[14]
*An. arabiensis*	LLDPE strands-DEET 30 %	78	2016	*	[14]
*An. arabiensis*	EVA strands-Icaridin 20 %	85	2016	*	[14]
*An. arabiensis*	EVA strands-Icaridin 30 %	82	2016	*	[14]
*A. aegypti*	DEET 10 %	> 83	2	*	[195]
*C. quinquefasciatus*	DEET 10 %	100	2	*	[195]
*A. albopictus*	DEET 24 %	> 90	6	*	[197]
*A. aegypti*	Exposis – Icaridin 25 %	-	10	Laboratório Osler do Brasil	[9]
*A. albopictus*	IR3535 20 % in ethanol solution	-	5	*	[205]
*A. aegypti*	IR3535 20 % in ethanol solution	-	> 9	*	[205]
*A. albopictus*	IR3535 10 %	-	> 7	*	[205]
*Anopheles dirus*	IR3535 10 %	-	8	*	[205]
*A. aegypti*	IR3535 10 %	-	> 6	*	[205]
*C. quinquefasciatus*	IR3535 10 %	-	8	*	[205]
*An. dirus*	IR3535 20 % in ethanol solution	-	> 3	*	[205]
*C. quinquefasciatus*	IR3535 20 % in ethanol solution	-	> 13	*	[205]
*C. tritaeniorhynchus*	IR3535 20 % in ethanol solution	-	> 14	*	[205]
*A. aegypti*	IR3535 10 % Spray®	95	6	*	[205]
*A. aegypti*	IR3535 10 % Spray®	90	6	*	[213]
*A. aegypti*	IR3535 10 % Spray®	85	7	*	[213]
*A. aegypti*	IR3535 15 % Spray®	95	6	*	[213]
*A. aegypti*	IR3535 15 % Spray®	90	6	*	[213]
*A. aegypti*	IR3535 15 % Spray®	85	6	*	[213]
*A. aegypti*	IR3535 10 % Lotion®	95	4	*	[213]
*A. aegypti*	IR3535 10 % Lotion®	90	5	*	[213]
*A. aegypti*	IR3535 10 % Lotion®	85	6	*	[213]
*A. aegypti*	IR3535 15 % Lotion®	95	6	*	[213]
*A. aegypti*	IR3535 15 % Lotion®	90	6	*	[213]
*A. aegypti*	IR3535 15 % Lotion®	85	6	*	[213]
*A. aegypti*	IR3535 20 % Spray®	95	6	*	[213]
*An. arabiensis*	IR3535 100 %	62	4	*	[10]
*An. arabiensis*	Blend IR3535 75 mol%-nonanoic	100	4	*	[10]
*A. aegypti*	IR3535 20 % Spray®	90	7	*	[213]
*A. aegypti*	IR3535 20 % Spray®	85	7	*	[213]
*Aedes spp. Culex spp.*	IR3535 20 % Pump spray	85	> 7	*	[214]
*Anopheles spp.*	IR3535 20 % Pump spray	-	> 7	*	[214]
*A. albopictus*	Icaridin 10 % Autan® spray	-	> 5	*	[200]
*A. aegypti*	Icaridin 10 % Lotion	95	6	*	[213]
*A. aegypti*	Icaridin 10 % Lotion	90	7	*	[213]
*A. aegypti*	Icaridin 10 % Lotion	85	8	*	[213]
*A. aegypti*	Icaridin 20 % Spray	95	6	*	[213]
*A.aegypti*	Icaridin 20 % Spray	90	7	*	[213]
*A. aegypti*	Icaridin 20 % Spray	85	9	*	[213]
*An. stephensi*	Bayrepel 20 % in complex solvent	100	8	*	[215]
*C. quinquefasciatus*	Bayrepel 20 % in complex solvent	100	8	*	[215]
*Culex annulirostris*	Icaridin® 19.2 % in ethanol Bayrepel Army®	≥ 99	5	*	[206]
*C. annulirostris*	Icaridin® 19.2 % in ethanol Bayrepel Army®	85	6	*	[206]
*Anopheles spp.*	Icaridin 19.2 % in ethanol Bayrepel Army®	>86	6	*	[206]
*Anopheles spp.*	Icaridin 19.2 % in ethanol Bayrepel Army®	>71	7	*	[206]
*An. arabiensis*	LLDPE strands-Icaridin 20 %	79	2016	*	[14]
*An. arabiensis*	LLDPE strands-Icaridin 30 %	98	2016	*	[14]
*An. arabiensis*	EVA strands-Icaridin 20 %	91	2016	*	[14]
*An. arabiensis*	EVA strands-Icaridin 30 %	88	2016	*	[14]
*A. albopictus*	Butyl anthranilate (BA) 0.1 %	> 53	-	*	[216]
*A. albopictus*	Ethyl anthranilate (EA) 0.1 %	> 38	-	*	[216]
*A. aegypti*	Ethyl anthranilate (EA) 10 %	100	-	*	[217]
*A. aegypti*	Ethyl anthranilate (EA) 5 %	90	-	*	[217]
*A. aegypti*	Ethyl anthranilate (EA) 2 %	78	-	*	[217]
*An. stephensi*	Ethyl anthranilate (EA) 10 %	96	-	*	[217]
*An. stephensi*	Ethyl anthranilate (EA) 5 %	80	-	*	[217]
*An. stephensi*	Ethyl anthranilate (EA) 2 %	68	-	*	[217]
*C. quinquefasciatus*	Ethyl anthranilate (EA) 10 %	88	-	*	[217]
*C. quinquefasciatus*	Ethyl anthranilate (EA) 5 %	82	-	*	[217]
*C. quinquefasciatus*	Ethyl anthranilate (EA) 2 %	64	-	*	[217]

^a^Poloxamer 407 is a triblock copolymer consisting of a central hydrophobic block of polypropylene glycol flanked by two hydrophilic blocks of polyethylene glycol (PEG)

### Polymer microcapsules as carriers of mosquito repellent

**Fig. 2:**
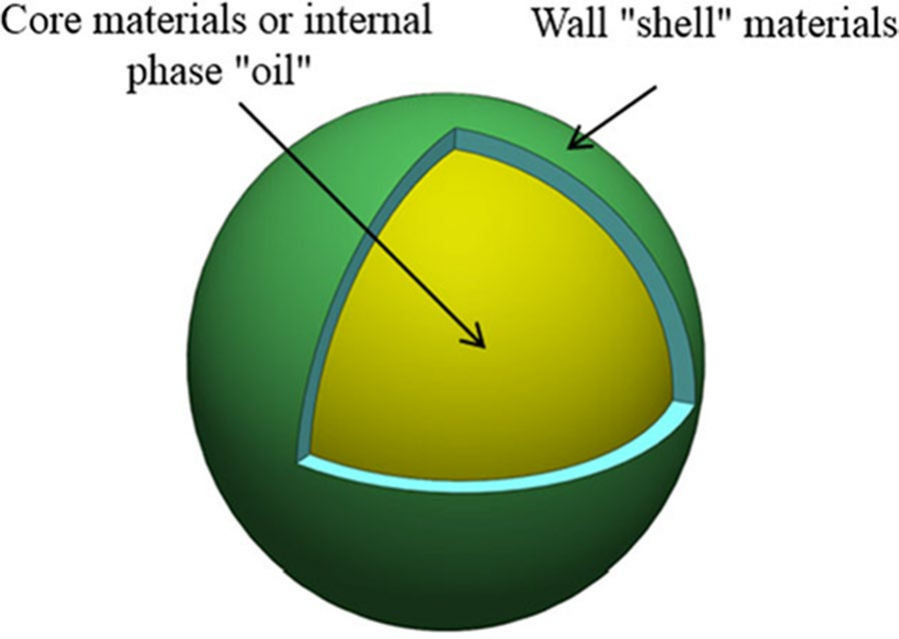
Example of a polymer microcapsule containing oil, where the core and wall or shell are clearly visible [26]. Republished with permission from reference

Polymer microcapsules are systems that consist of a functional barrier between core material containing the active ingredient, e.g. repellent, as an internal phase, and a wall (natural or synthetic polymer) also known as a shell or a membrane (Fig. 2) [26]. In order to adapt to the several types of core and wall materials as well as to produce different particle sizes, shell thicknesses and permeabilities, hence, adjusting the release rate of active ingredient [27], several methods can be used to prepare polymer microcapsules. The common methods include spray drying [27–29], interfacial polymerization [30], and coacervation [31–34].

#### Mechanism of release of repellent polymer microcapsules

**Fig. 3:**
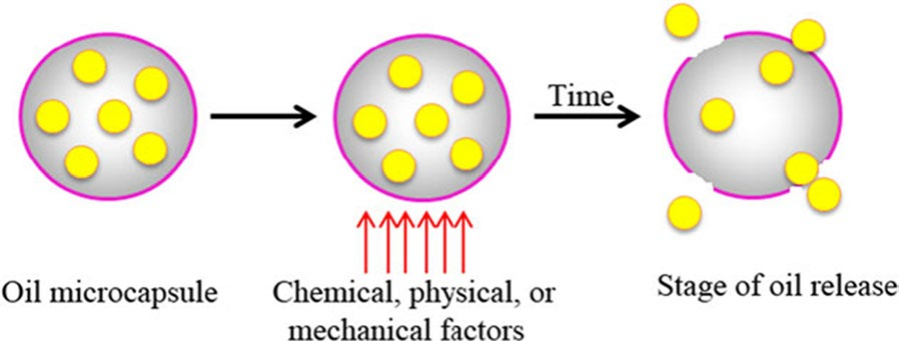
Example of an oil controlled-release mechanism through a polymer microcapsule wall [26]. Republished with permission from reference

The release of active ingredient (repellent) is controlled by diffusion and volatilization from polymer microcapsules following disruption or rupture of the structure of these carriers after skin application [35, 36]. A schematic diagram of controlled oil release through microcapsules is presented in Fig. 3. The cavity of polymer microcapsules is sufficiently large for storage of the volatile repellent. The uniform thickness and layer of a polymer wall or shell resists the diffusion of repellent, providing controlled-release and extending their effectivness under desired conditions. It also determines the stability of the microparticles and the level of protection of the core material against chemical, physical or mechanical attacks [26, 35]. Additionally, the microcapsules demonstrated being suitable formulations to reduce the volatility of repellents, extending the protection time [33, 35, 37, 38].

**Fig. 4:**
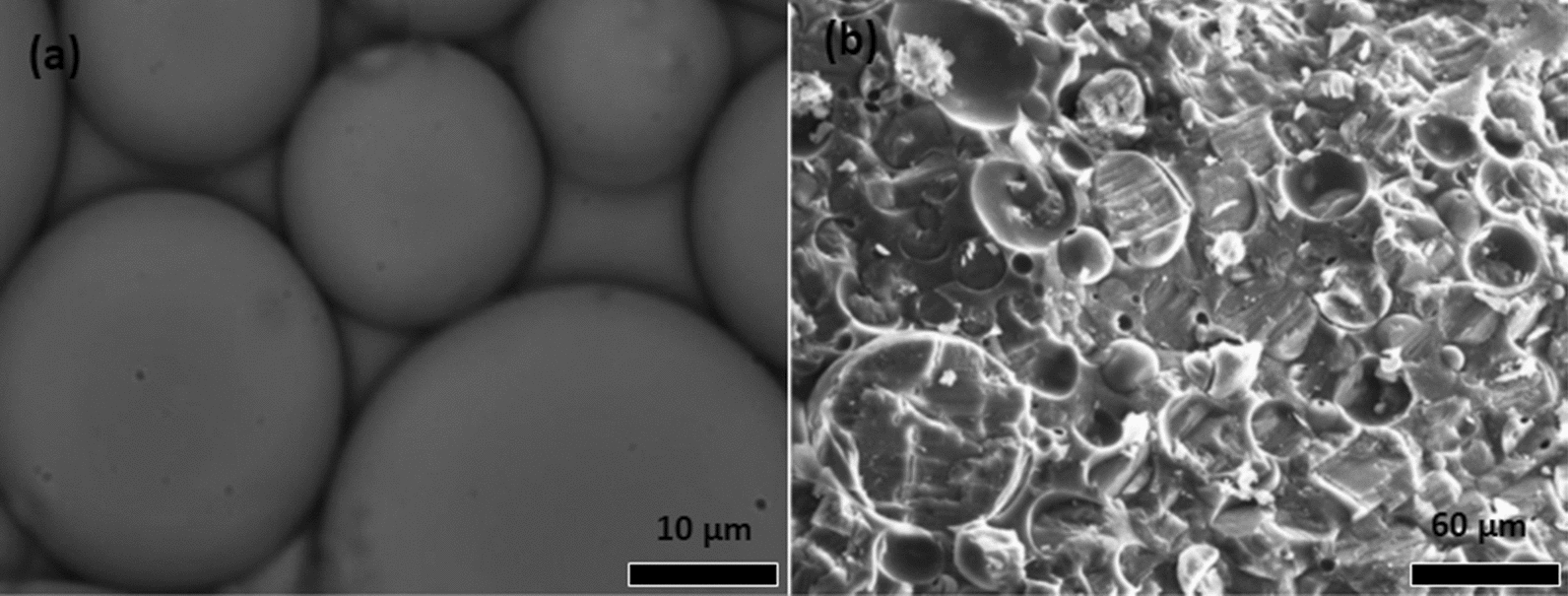
Optical (**a**) and SEM (**b**) micrographs of thyme oil/poly(lactic acid) microcapsules [39]. Republished with permission from Taylor & Francis

**Fig. 5:**
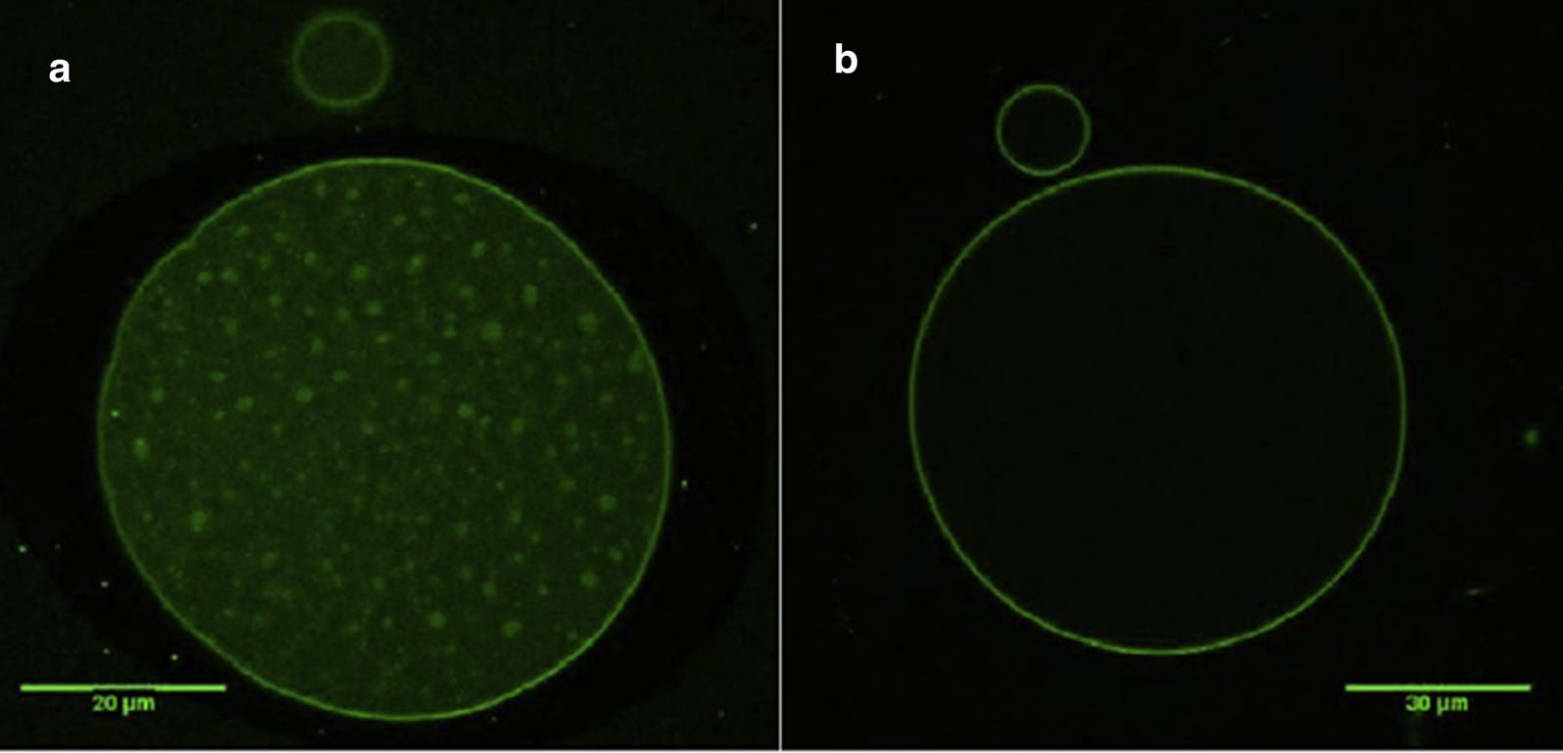
Fluorescence confocal micrographs of the thyme oil/PLA microcapsules: **a** Core and wall are clear visible; while **b** wall or shell polymer is visible [39]. Republished with permission from Taylor & Francis

The morphology of polymer microcapsules containing repellent has been evaluated by microscopy [27]. For example, the morphology-features of polylactic acid microcapsules containing thyme oil was analysed by optical microscopy (Fig. 4a) and scanning electron microscopy (Fig. 4b) [39]. The optical micrograph shows droplets of oil encapsulated, suggesting that the particles are spherical without noticeable agglomeration. The SEM micrograph confirmed the irregular surfaces of PLA microcapsules with small holes and pores. Additionally, the presence of an outer membrane of PLA that covers the oil was confirmed by confocal microscopy (Fig. 5) [39].

For mosquito control, studies showed that the repellent-based microcapsules offer an alternative to increase the period of protection against mosquitoes while avoiding the contact of the mosquitoes with the skin [32]. For instance, a study by Specos et al. [40] confirmed that polymer microcapsules with oil of Citriodiol® (from Citrefine International Ltd.) exhibited a controlled-release of repellent and was effective against *Ae. aegypti* for more than 30 days. N,N-Diethyl-3-methylbenzamide (DEET)-based polymer microcapsules presented a slow release of DEET for a long period of time and likewise diminished its skin permeation [38, 41]. It was also effective against *Aedes albopictus*. Furthermore, citronella-oil microcapsules exhibited a long protection time against *Ae. aegypti* for three weeks [32]. Those studies show the relationship between the release rate and long-duration protection of repellents loaded into microcapsules against mosquitoes.

#### Stability study of the polymer microcapsules as carriers of mosquito repellent

The microencapsulation technique provides stability to the mosquito repellents, allowing their controlled release under certain conditions [42]. It is, therefore, key to consider on improving the stability of the repellents, if they are to be used as active ingredients in the development of repellent-based formulations so that the level of effectiveness in the final use is a good. For example, Bezerra et al. [43] evaluated the thermal stability of the repellents-based on microcapsules formulations. The results of the thermal analysis revealed that incorporation of citronella essential oil by the microencapsulation process resulted in a complex with high thermal stability compared with the free oil, indicating that microencapsulation protects the oil, making it more resistant to evaporation. More studies about the stability of repellents based on polymer microcapsules were also reported by reserchers [28, 44].

## Nanoemulsions as carriers of mosquito repellent

**Fig. 6:**
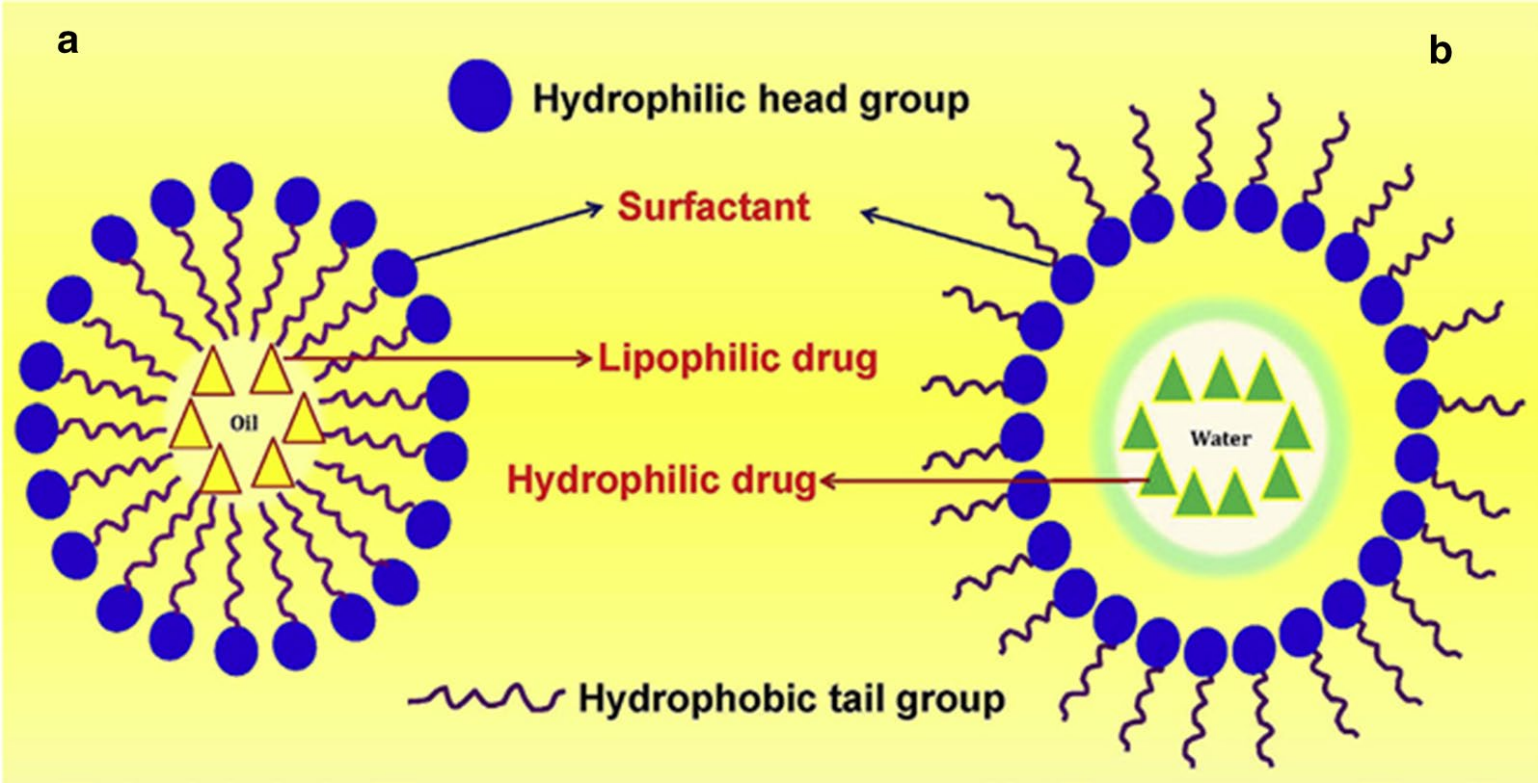
Nanoemulsion structure comprising: **a** oil-in-water emulsion (left), and **b** water-in-oil emulsion (right) [49]. Republished with permission from Elsevier

Nanoemulsions are kinetically stable systems consisting of oil and water dispersions stabilized with surfactants [45–48]. Figure 6 shows the structure of a typical nanoemulsion [49]. Such systems can be prepared by high energy and low-energy methods [46, 50].

### Pseudo‐ternary phase diagram

**Fig. 7:**
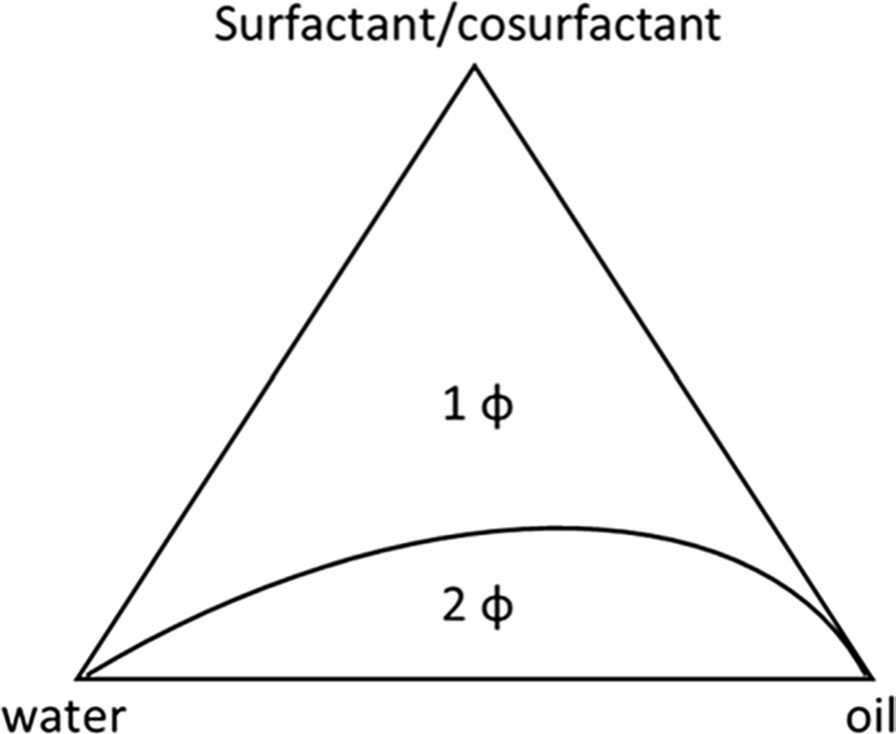
Example of a pseudo-ternary phase diagram of a simple four-component microemulsion (surfactant, cosurfactant, oil and water), at constant temperature and pressure. (1 ɸ): one phase; (2 ɸ): two phases [51]. Republished with permission from Elsevier

A nanomeulsion device comprises a boundary between the oil and water phases at which the surfactant is located. Those systems are typically formed only in a specific and narrow range of concentrations for a given surfactant-oil-water structure. The relationship between the phase behaviour of different mixtures and their composition is generally depicted using a pseudo-ternary phase diagram (Fig. 7), where a corner represents a binary mixture of surfactant-co-surfactant, water-drug and oil-repellent. Outside the nanoemulsion region, the amount of surfactant is too low to allow the formation of a single nanoemulsion phase, therefore leading to the existence of multiphase systems [51].

Based on the components, nanoemulsions are divided in: (i) oil-in-water (O-W) (the oil phase is dispersed into a continuous water phase); (ii) water-in-oil (W-O) (the water phase is dispersed in a continuous oil-phase), and, (iii) bi-continuous or multiple emulsions in which micro domains of phases of the oil and water are interdispersed [45].

**Fig. 8:**
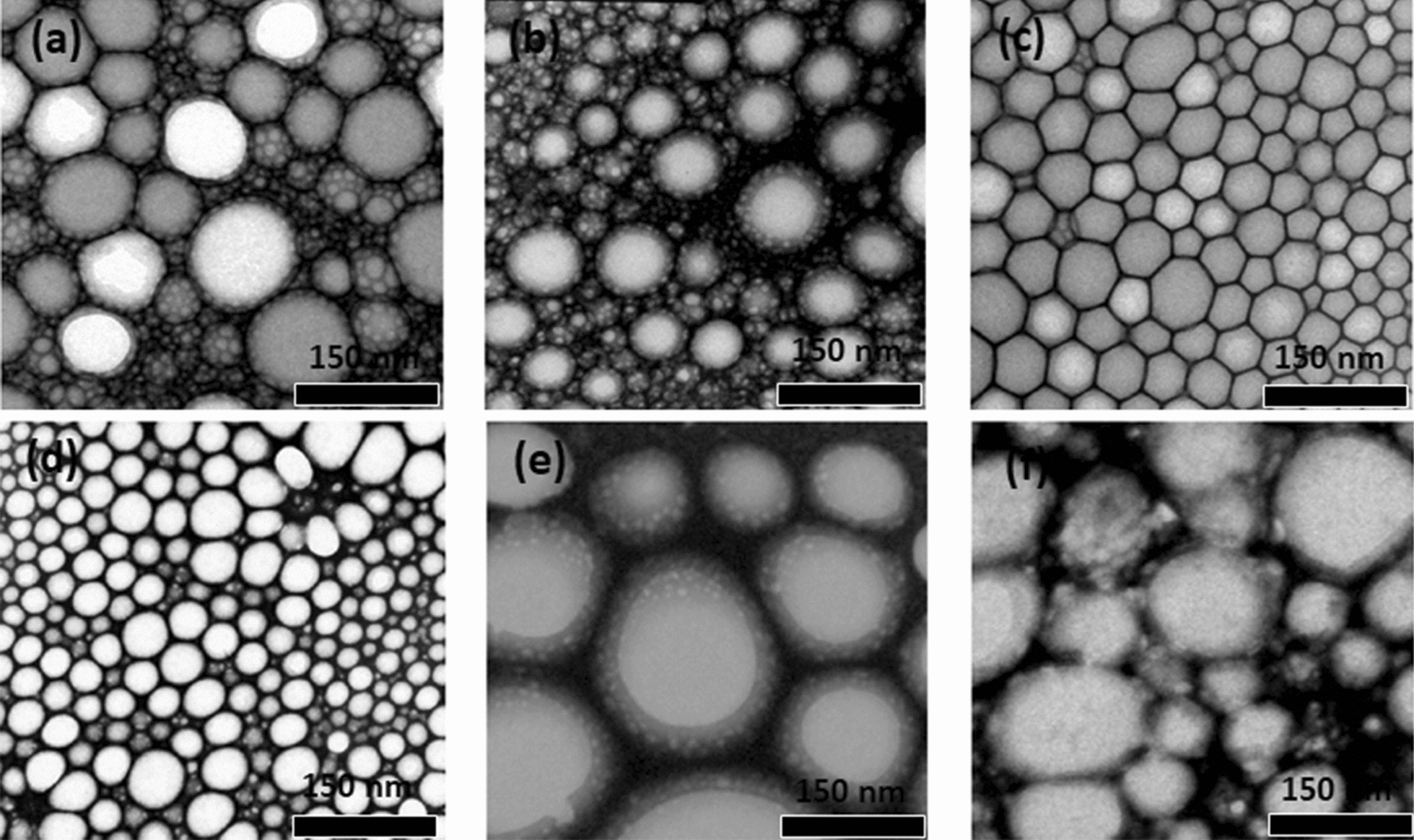
TEM micrographs of nanoemulsions with several oils: **a** phenyl trimethicone, **b** polydimethylsiloxane, **c** cetyl ethylhexanoate, **d** dioctanoyl-decanoyl-glycerol, **e** isopropyl myristate, and **f** liquid paraffin. Scale bars: 150 nm [48]. Republished with permission from American Chemical Society (ACS)

**Fig. 9:**
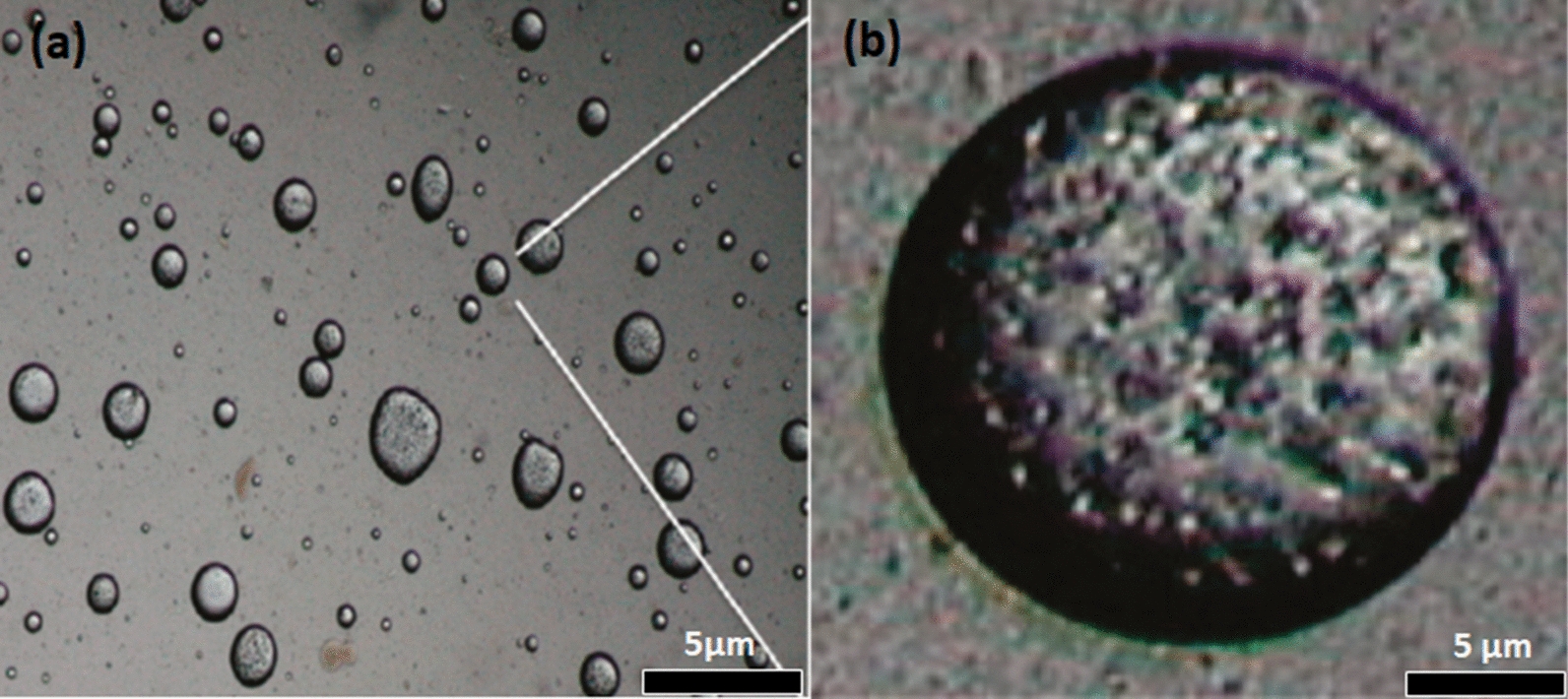
Optical micrographs of the oil-in-water embryonic emulsions containing a 1:10 mixture of blend poly(ethylene oxide)/poly(ε-caprolactone) and phenyl trimethicone oil in the organic phase. Scale bars: 5 μm [48]. Republished with permission from American Chemical Society (ACS)

The nanoemulsion morphology has been obtained by microscopy [52]. As an example, the surface morphology of nanoemulsions stabilized by semi-solid polymer interphases were investigated by transmision electron microscopy (TEM) as is presented in Fig. 8 [48]. The results showed that the type of oil affected the morphology of nanoemulsions. In addition, the optical micrographs of a nanoemulsion confirmed that the resulting droplet size was in the range of a few micrometres (Fig. 9) [48].

**Fig. 10:**
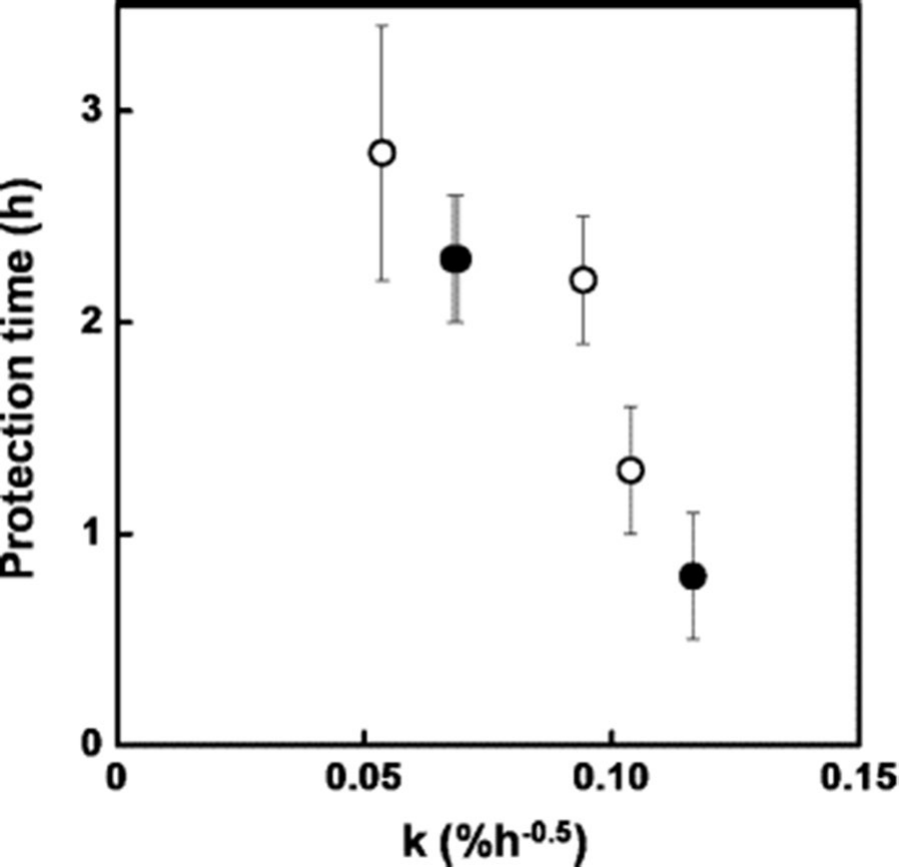
Relationship of protection time and release rate of citronella oil in a nanoemulsion at varying concentration of glycerol (black circle) and surfactant (white circle) [56]. Republished with permission from Elsevier

Due to problems caused by classic formulations, that is, solutions and lotions, available on the market including irritation and skin dryness when applied on the human skin, the nanoemulsions of repellents are promising systems for reducing mosquito-borne diseases [35]. This may be explained by their intrinsic physicochemical properties, such as, uniform and very small droplet sizes (20–200 nm), low viscosity and optical transparency [53]. Finally, several works have reported the activity of nanoemulsified repellents against mosquitoes, boosting in this way their use in the control of infectious diseases [53]. For example, a study done by Sugumar et al. [54] reported a good performance of eucalyptus oil nanoemulsion against the mosquito *Culex quinquefasciatus*, where the activity of essential oil nanoemulsions was related to the size of the oily droplets. Nuchuchua et al. [55] also reported that the small oily-droplet size displays an important role in the efficacy of oil nanoemsulsions. Specifically a study conducted by Anjali et al. [50] showed that the small size of the neem seed oil nanoemulsion was much more effective against *C. quinquefasciatus* when compared to oil nanoemulsions with a medium and large sizes. Sakulku et al. [56] showed the effect of citronella oil nanoemulsion against *A. aegypti*. The results of protection time was directly related to the release rate of citronella oil from the nanoemulsion as is shown in Fig. 10. Furthermore, the effect of emulsification on the release rate of citronella oil aiming to understand the relationship between droplet size and the release rate was also investigated by Agrawal et al. [57]. Release rate studies of samples with different droplet diameters of 65 nm and 72 nm but the same composition was carried out at 35 ^o^C. It was found that the evaporation rate was high for a sample with small droplets compared to large droplets. Similar behaviour was observed by others [55, 56].

Due to the small size and low polydispersity of droplets in nanoemulsions, those have advantages over other repellents-carrier systems [58]. The advantages include: (i) better physical stability against creaming, flocculation, sedimentation, and *Ostwald ripening* than ordinary emulsions; (ii) efficient permeation ability and enhanced bioavailability because of high surface area/volume ratios and small droplet size, thereby enhancing the transfer of molecules through biological membranes; (iii) improved water solubility, tunable loading capability and enhanced chemical stability; (iv) slow release of bioactive compounds through encapsulation and solubilization, and (v) low dosage of emulsifiers, compared with microemulsions (the dosage of emulsifiers in microemulsions is roughly ≥ 20 % while it is between 2 and 10 % in nanoemulsions) [58–60].

### Stability of the nanoemulsions as carriers of mosquito repellent

As previously reported, the nanoemulsions present several advantages due to the small droplets size they contain, high optical clarity, good physical stability and droplet aggregation, and enhanced bioavailability of encapsulated substances, which make them suitable for final applications [59]. Depending on desired formulation, preparation method should be selected to optimize droplet size distribution since it strongly affects stability behaviour. Therefore, systems with droplets diameter smaller than 200 nm and a monomodal distribution usually have a homogeneous structure, that is, a structure with well distributed droplets that do not show flocs [59]. A structure with these characteristics remains unchanged for long time, up to six months, given the nanoemulsion enhanced stability compared to conventional emulsions with the same formulation [59]. Therefore, the development of systems with potential technological application also requires the existence of long-term stability. For example, Lucia et al. [61] evaluated the stability of the emulsions after an aging of 28 months. The results demonstrated that the essential oils based on nanoemulsions before and after aging showed that samples containing eugenol oil as main component did not present any significant change on their homogeneity after 28 months of aging, maintaining their monodisperse character and a constant droplets size. The findings on the stability of essential oils based on nanoemulsion could be considered a promising pre-requisite to the development plant-derived repellents formulations against mosquitos.

## Solid lipid nanoparticles as carriers of mosquito repellent

**Fig. 11:**
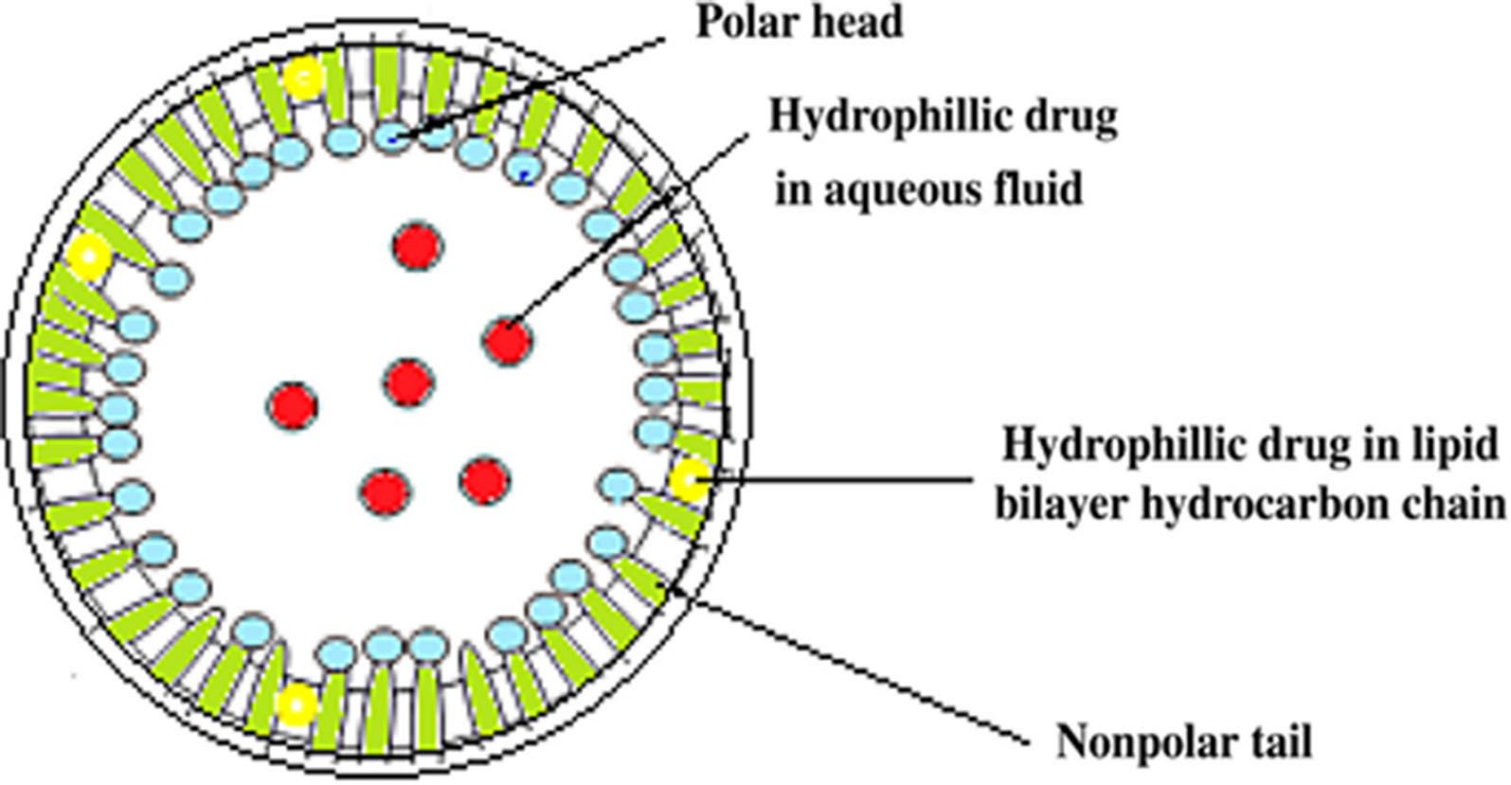
Solid lipid nanoparticle structure [62]. Republished with permission from Elsevier

Solid lipid nanoparticles (SLN) gained the attention of researchers because oftheir excellent features that include high surface area, high capacity of drug loading, high stability of repellent, and high capacity to incorporate feasible lipophilic and hydrophilic repellent. Their basic components consist of lipids and emulsifiers [62, 63]. Lipids are the essential components in SLN formulations because they control the stability, release, encapsulation and loading capacity [62]. The emulsifiers are used to stabilize the lipid dispersion. Studies report that the combination of emulsifiers can prevent particle agglomeration more efficiently. A clear advantage of solid lipid nanoparticles is the fact that the lipid matrix is made from physiological lipids, which reduces the the danger of acute and chronic toxicity [63]. Figure 11 shows the structure of an SLN system where the hydrophilic and hydrophobic drugs can be encapsulated in polar head and nonpolar tail lipid chains [62].

**Fig. 12:**
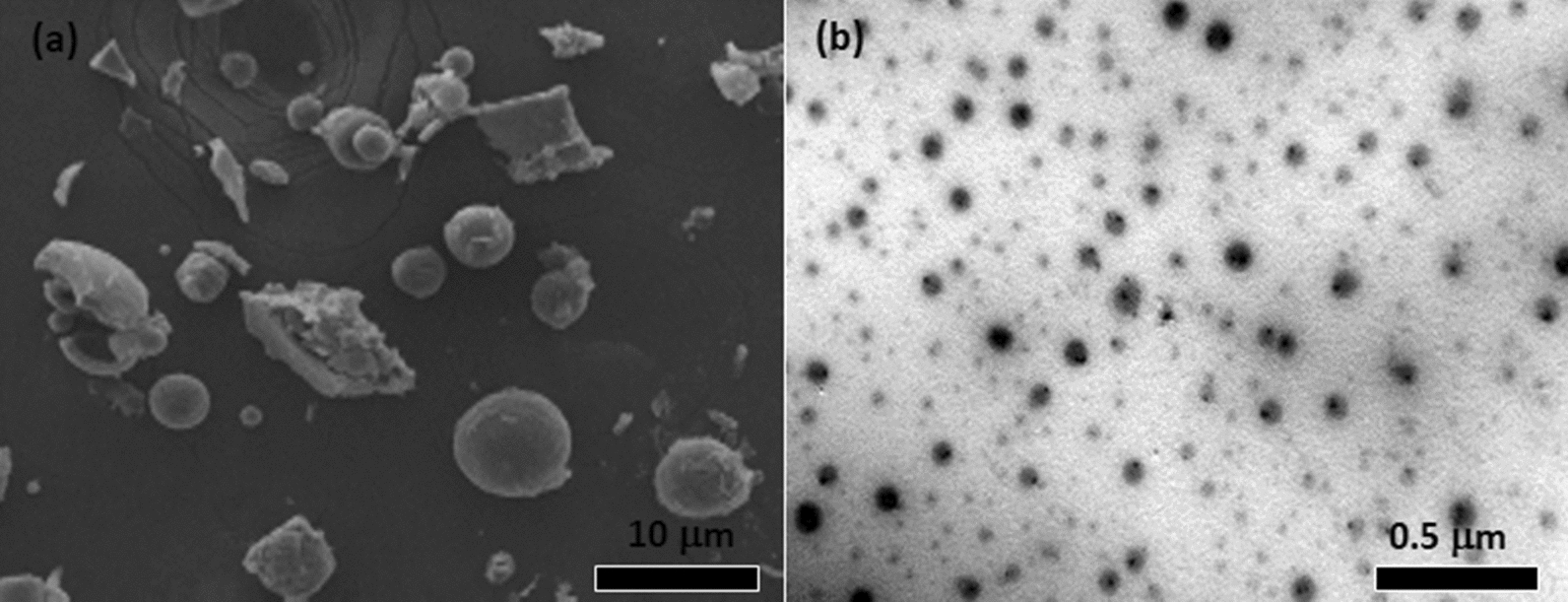
**a** SEM micrograph of neem seed oil loaded solid lipid nanoparticles [65] and **b** TEM micrograph of essential oil into solid lipid nanoparticles [66]. Republished with permission from Elsevier

The morphology of SLNs along with their particle size and distribution can be assessed by electron microscopy [64]. For example, the surface morphology of neem seed oil incorporated into solid lipid nanoparticles was visualized by SEM, where spherical particles with a smooth surface were observed (Fig. 12a) [65]. Further, Adel et al. [66] evaluated the morphology of geranium oil loaded SLNs by TEM where the particles appeared round/spherical in shape, having a good dispersion and narrow size (Fig. 12b).

### Mechanism of repellent release from solid lipid nanoparticles (SLNs)

The mechanisms of repellent release are diffusion, degradation, and swelling followed by diffusion. Solid lipid nanoparticles may display any or all of these mechanisms of release of repellents. Diffusion can occur across the lipid matrix on a macroscopic scale due to pores therein or by passing through lipid chains on a microscopic level. The repellent diffusion rate through SLNs typically reduces with time since initially repellent diffuses from the surface of particles to release media followed by diffusion from interior layers. Consequently, repellent moves gradually slower and needs an extended diffusion time to release [62]. Wissing et al. [67] reported that the drug release through SLNs systems is influenced by modification of the lipid matrix, surfactant concentration and production parameters.

Repellents based on solid lipid nanoparticles have demonstrated a good potential for insect control. Studies reported various essentials oils into SLNs as repellent-based products against mosquitoes [68, 69]. For example, Iscan et al. [70] reported that DEET-loaded solid lipid nanoparticles controlled the release of repellent and its skin permeation. Other works also showed that the repellent-based solid lipid nanoparticles are suitable carriers for pest control. A study conducted by Lai et al. [71] demonstrated that essential oil incorporated into SLNs controlled the evaporation rate of oil over 48 h at 35 ^o^C during storage. Furthermore, the performance of garlic essential oil loaded SLNs against the Red flour beetle *Tribolium castaneum* maintained effective repellence over five months, because the active ingredient continued to be released slowly through particles [72]. This suggests that it is possible to incorporate garlic essential oil into polyethylene glycol (PEG) coated nanoparticles to control pests that affect the stored products. Finally, those studies showed that SLNs systems show the capacity to reduce quick volatility and degradation of repellents, it also improves stability and keeps the minimum effective amount needed and facilitates their use.

### Stability of the solid lipid nanoparticles as carriers of mosquito repellent

Solid lipid nanoparticles (SLNs) have emerged as an alternative to other novel delivery approaches due to various advantages such as feasibility of incorporation of lipophilic and hydrophilic drugs and improved stability [73]. For example, Lai et al. [71] incorporated *Artemisia arborescens* essential oil into solid lipid nanoparticles by high-pressure homogenization method. The obtained *Artemisia arborescens* essential oil-loaded SLNs demonstrated high physical stability at various storage temperatures over 2 months, and they were able to reduce the rapid evaporation of essential oil during *in vitro* release experiments compared with reference emulsions. Furthermore, Tian et al. [74] evaluated the thermal stability and chemical stability of citral oil-loaded solid lipid nanoparticles. The results demonstrated that the thermal stability of citral improved when it was encapsulated in the citral-SLNs. In terms of chemical stability of citral in the citral-SLNs formulation remainaded stable during 12 days of storage at 37 °C. From the results, it can be said that the citral-SLNs could be used as a carrier to effectively protect citral from degradation in acidic environments.

## Polymer micelles as carriers of insect repellent

**Fig. 13:**
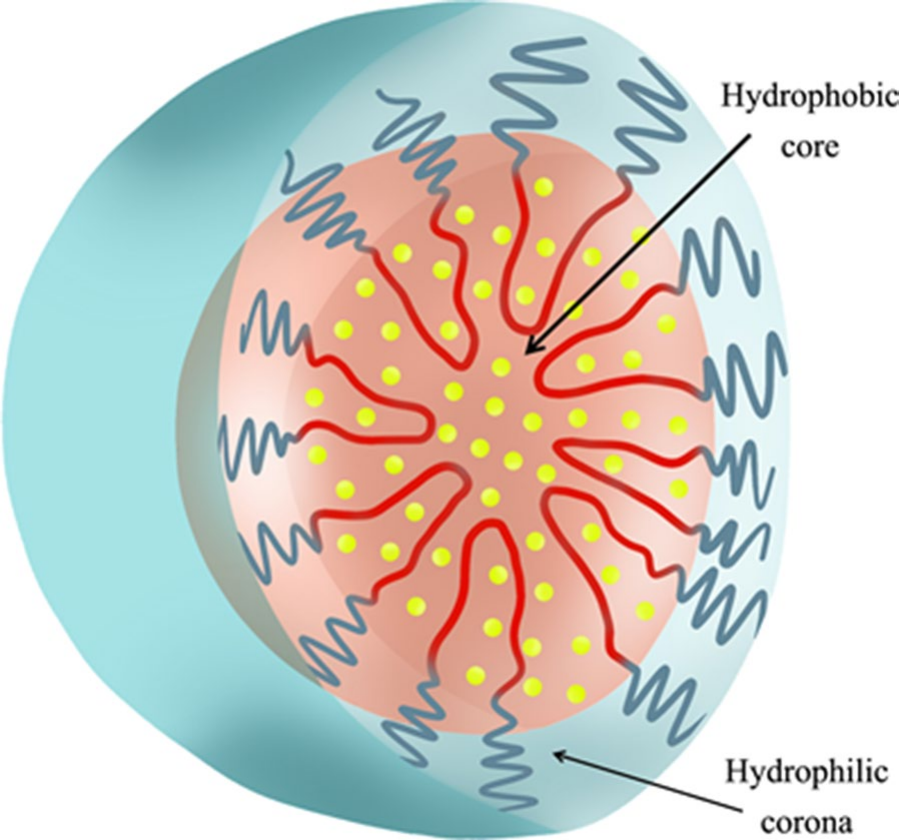
Structure of repellent (essential oil) based polymer micelles [79]. Republished with permission from reference

Polymeric micelles comprise two regions known as the core and the shell (Fig. 13). The internal core represents a hydrophobic region of a block copolymer, which encapsulates the poorly water-soluble repellent, while the external region of the copolymer hydrophilic block, known as the shell or corona, defends the drug from the aqueous environment [75].

### Mechanism of repellent release from polymer micelles

The repellent loading can be done by chemical conjugation or physical entrapment [75]. In the chemically conjugated repellent, the release rate of repellent occurs by bulk degradation of the polymer matrix or surface erosion [75]. Physically, for the repellent entrapped into polymeric micelle, the repellent release is controlled by diffusion through the core of the polymeric micelle, stability of micelles and the copolymer degradation rate. If the polymeric micelle system is stable and the polymer degradation rate is slow, the repellent difussion rate is determined by the compatiblity between the drug and core forming block copolymer, the quantity of repellent entrapped, the repellent molecular volume, and length of the core forming block polymer [76].

**Fig. 14:**
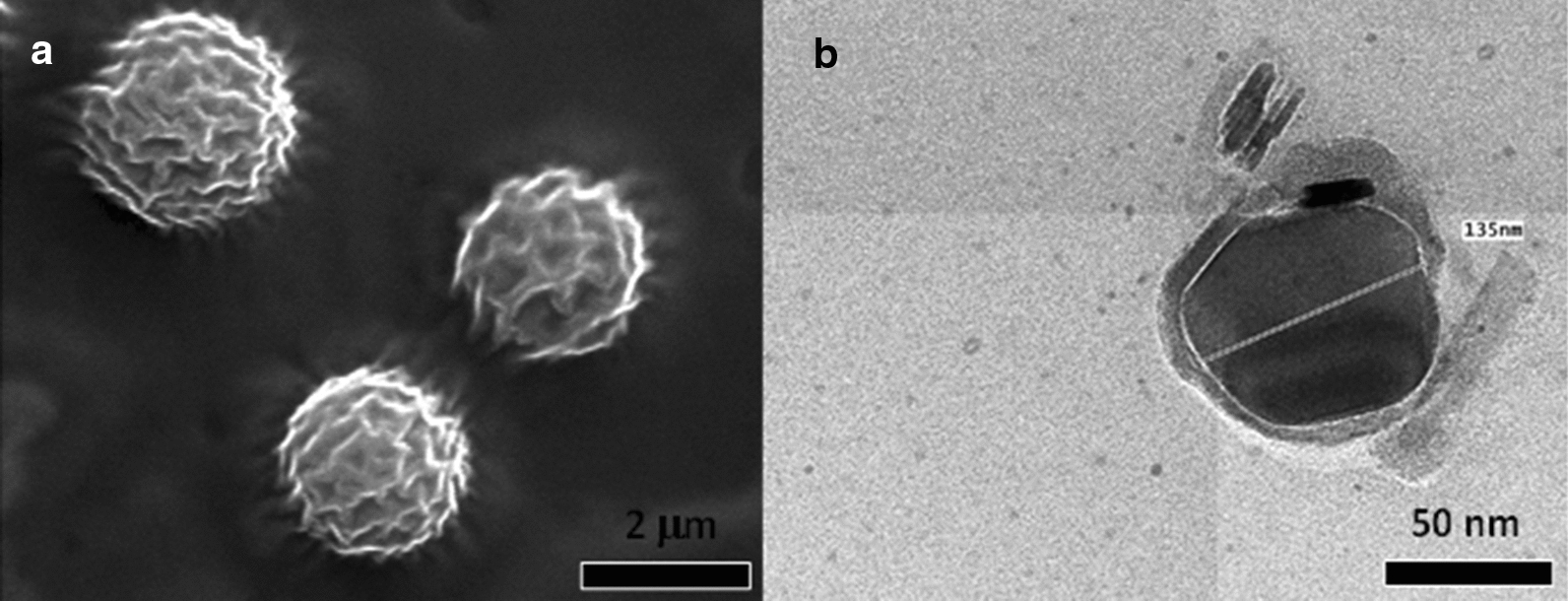
**a** SEM micrographs of DEPA-based micellar- polymer system. The presence of a dense polymeric sheath of polymer was confirmed. **b** TEM micrographs of DEPA-based micellar-polymer system. The presence of a discontinuous polymeric layer was revealed [78]. Republished with permission from Elsevier

Polymeric micelles can be used as mosquito repellent carrier-systems as well as for guided release due to their high encapsulation capacity [77]. A work done by Barradas et al. [77] reported that DEET placed into micellar formulations based on poly(ethylene oxide)–poly(propylene oxide)–poly(ethylene oxide) triblock copolymer exhibited slow release of DEET during up to seven hours. Therefore, there was a reduction of approximately 35 % in the amount of DEET absorbed through the skin after six hours. Balaji et al. [78] encapsulated an insect repellent, diethylphenylacetamide (DEPA) in polymeric nanomicelles and evaluated their performance against *Culex tritaeniorhynchus*. The median lethal concentration (48 h) for third instar *C. tritaeniorhynchus* larvae were found to be 0.416 mg/L for bulk DEPA and 0.052 mg/L for nano DEPA. The core-shell structure of the micelles was confirmed by SEM (Fig. 14a) and TEM (Fig. 14b).

Other studies reported the activity of polymeric micelle-based repellents for insect pest control. For example, Lucia e*t al.* [79] evaluated essential oil components (EOCs) based polymeric micelles system against against *Pediculus humanus capitits (Head lice)*, where the product was effective with a mortality above 60 %, being the most effective system containing linalool, 1,8-cineole, terpineol, thymol, eugenol, and geraniol. Therefore, the study suggested that essential oil-based polymeric micelles are alternative tools for insect control.

## Cyclodextrins as carriers of mosquito repellent

**Fig. 15:**
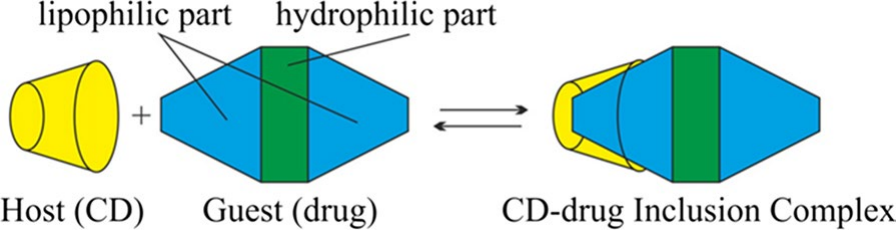
An example of drug (i.e. repellent) encapsulation in cyclodextrin at a 1:1 ratio [81]. Republished with permission from Elsevier

Cyclodextrins are cyclic oligosaccharides _D_-glucopyranose based with a hydrophilic outer surface and a hydrophobic central cavity [35]. Based on the number of _D_-glucopyranose units, there are three main types of cyclodextrins classified as (i) α-cyclodextrin (six units), (ii) ß-cyclodextrin (seven units), and (iii) γ-cyclodextrin (eight units) [35]. Among the mentioned cyclodexitrins, ß-cyclodextrin (ß-CD) is most often commercially available and used by researchers due its easy production and low cost. The internal region of the cavity of the ß-cyclodextrin sufficient to accommodate a mosquito repellent molecule [80]. Figure 15 shows the formation of the 1:1 inclusion complex [81]. Inclusion complexes are compounds with the characteristic structure of an adduct, where one compound (host molecule) spatially encloses another. The enclosed compound (guest molecule) is situated in the cavity of the host without much influencing of the host framework structure. Apart from a slight deformation, it is a characteristic feature that the size and shape of the available cavity stays basically unchanged [82].

**Fig. 16:**
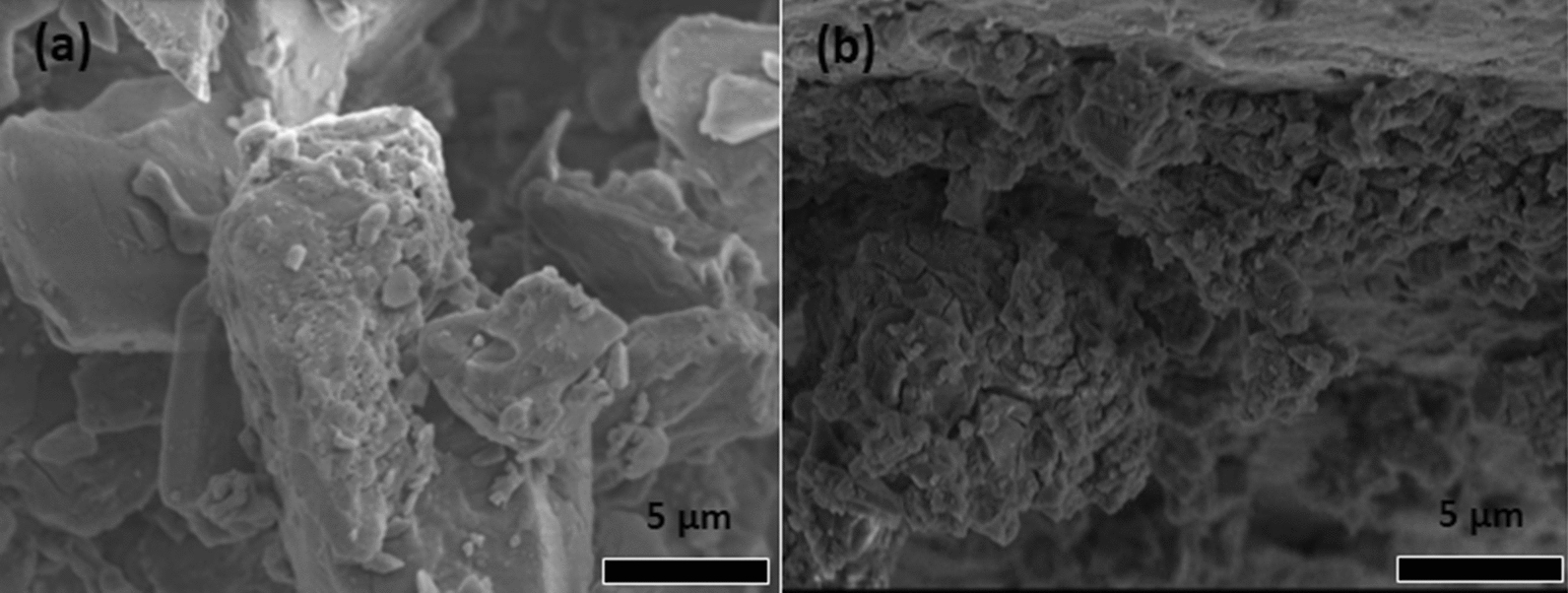
SEM micrographs of **a** ß-cyclodextrin and repellent essential oil based on ß-cyclodextrin inclusion complex [83]. Republished with permission from Elsevier

The formation of a ß-cyclodextran inclusion complex with repellent (essential oil) was confirmed by SEM micrographs by the presence of changes of the particle shape (Fig. 16b) compared to SEM micrographs of ß-cyclodextran (Fig. 16a) [83].

Cyclodextrins are commonly listed as generally recognized as safe by the Food and Drug Administration (FDA) [84, 85]. Repellents present an ideal characteristic for complexation with cyclodextrins because they are small, hydrophobic molecules. Cyclodextrin formulations are suitable to entrap mosquito repellents in which it diminishes the repellent evaporation rate, increasing their performance for a long period of time [35]. Studies showed that repellent-based cyclodextrins are effective against mosquitoes. For example, a study by Hebeish et al. [80] evaluated the limonene oil-β-cyclodextrin (β-CD) formulation against mosquitoes. The results showed that the repellency, knockdown and killing against/of mosquitoes increased with the concentration of limonene in cyclodextrin-finished cotton fabrics within the range studied (250–1500 mg/m^2^). Furthermore, it was found that the activity of repellent-based products increased with increasing exposure time. Finally, the authors reported that the treated fabric can be washed and stored while keeping their repellency activity property.

Songkro et al. [86] reported that the release rate of citronella oil from the lotions containing the inclusion complexes was lower than that of the prepared lotion containing normal citronella oil. Additionally, the repellency activity againt *Ae. aegypti* was also observed in the device with the citronella oil–ß-cyclodextrin inclusion complex. These results suggest a controlled release of the citronella oil from the inclusion complex. Galvão et al. [83] showed that the *Citrus sinensis* essential oil (CSEO) from the oil-ß-cyclodextrin inclusion complex, exhibited excellent larvicidal activity with 100 % mortality of *Ae. aegypti* larvae after 24 h. The complexation of essential oils with β-cyclodextrin has been much investigated. The studies showed that the volatility of the repellent can be released slowly from β-cyclodextrin, consequently increasing the duration time of the product against insects.

### Stability of the cyclodextrins as carriers of mosquito repellent

Stability studies ensuring the maintenance of product quality, safety and efficacy throughout the shelf life are considered as pre-requisite for the acceptance and approval of any pharmaceutical product. These studies are required to be conducted in a planned way following the guidelines issued by the WHO and or other agencies [87]. For example, Bezerra et al. [88] investigated the thermal stability of the complexes using the thermogravimetric analysis (TGA). The authors found that the citronella oil-ß-cyclodextrin inclusion complex presented higher thermal stability and lower presence of water. This fact has happened due to the encapsulation of citronella oil, which came to occupy the place previously taken by the molecules of water, as described. Finally, with the increase in temperature, the oil is released with the decomposition of the biopolymer that protects it. On the other hand, the thermal stability of the oil was improved.

## Liposomes as carriers of insect repellent

Liposomes are vesicular structures formed by a hydrophilic aqueous core and a lipophilic phospholipids bilayer. Due to their composition, they are biocompatible and excellent for administering repellents to the skin [35]. They are capable of delivering hydrophilic and lipophilic repellent into their compartments. When applied to the skin, they form a prolonged release reservoir systems. Liposomes are considered ideal formulations to carry repellents due the advantages of reduced evaporation rate, extended release, improved action time duration, low skin permeation and toxicity [35]. Studies done by researchers [89–91] demonstrated that the liposome systems with the repellents DEET and essential oil, offered sustained release of active ingredients, improving their activity against insects even up to 48 h. There are few works in the literature that report liposomes containing insect repellents. However, more studies about preparation of new formulations based on repellents-liposomes for the control of mosquitoes are required.

### Stability of the liposomes as carriers of insect repellent

Several studies have evaluated the of stability of liposomes formulations. Example, Valenti et al. [90] evaluated the stability of the liposome-incorporated *Santolina insularis* essential oil (EO). The kinetic study of the stability of liposomes encapsulating EOs was monitored in terms of liposome sizes, encapsulation efficiencies and zeta potential measurements. All prepared liposomal formulations showed a very good stability for more than one year, when stored at 4–5 °C. Therefore, the results obtained showed that at least 93–96 % of the incorporated oil was already associated with liposomes after one year. Dynamic light scattering measurements showed that average size and size distribution remained constant for at least one year. Moreover, the liposomes were able to prevent essential oil degradation compared to the neat oil. In summary, the liposome incorporation protected the essential oil from degradation and after one year its composition was still very close to that of the freshly prepared oil. A minimal loss of the more volatile compounds was observed and no identifiable degradation product was present even in traces. Lastly, it had been reported that *Eucalyptus camaldulensis* EO liposome had minor changes in the size and it remained stable after three (3) months of storage [92]. Additionally, more studies about the stability of liposomes encapsulating EOs were also investigated by authors [93–96].

## Microporous polymers as carriers of insect repellent

Microporous polymer structures have been prepared by different methods, including non-solvent-induced phase separation (NIPS) [97], solvent-induced phase separation (SIPS) [98], thermally induced phase separation (TIPS) [99] and thermally assisted evaporation phase separation (TAEPS) [100]. Among all the methods mentioned above, TIPS introduced by Castro [99] has become the most useful technique for the preparation of microporous polymer structures [101, 102]. Due to its advantages such as ease of control and a low tendency to produce defects, TIPS is able to produce a variety of relatively thick isotropic microporous microstructures capable of producing a suitable controlled release [103, 104].

Microporous materials gained most interest in several potential membrane applications in the fields of microfiltration, ultrafiltration, reverse osmosis, gas separation, clean energy, catalysis and storage media due to their extraordinary high porosity and surface area [105]. Various solvents such as dioxane/water, chloroform, methanol, ethanol, hexane, or dichloromethane with polymers such as (i) polypropylene (PP) [102, 106–108], polyvinylidene fluoride (PVDF) [109–112], (iii) poly(ethylene-co-vinyl alcohol) (EVOH) [113, 114], (iv) polystyrene [115, 116], (v) polyethylene (PE) [101, 102, 106, 117–122], or poly (lactic acid) [123] have been used to form microporous structures through the TIPS method. Therefore, the preparation of microporous structures using polyolefins is widely explored due to their good thermal and solvent resistance as well as their low cost. Generally, to prepare the microporous polymer structures by the TIPS method, the steps listed below are followed [101, 102, 118]: (i) a solution is obtained at a high temperature through mixture of the polymer with liquid (i.e. repellent). The liquid is mostly a low molecular weight and high-boiling-point diluent in which the polymer is actually insoluble at room temperature [117]; (ii) the solution is then rapidly cooled or quenched to induce solid-liquid or liquid-liquid (L-L) phase separation; (iii) the liquid is extracted from the polymer device by an appropriate solvent or by evaporation (at ambient temperature), and finally, (iii) a microporous polymer with the desired structure is formed.

### Phase diagram of a typical miscible polymer‐repellent system

**Fig. 17:**
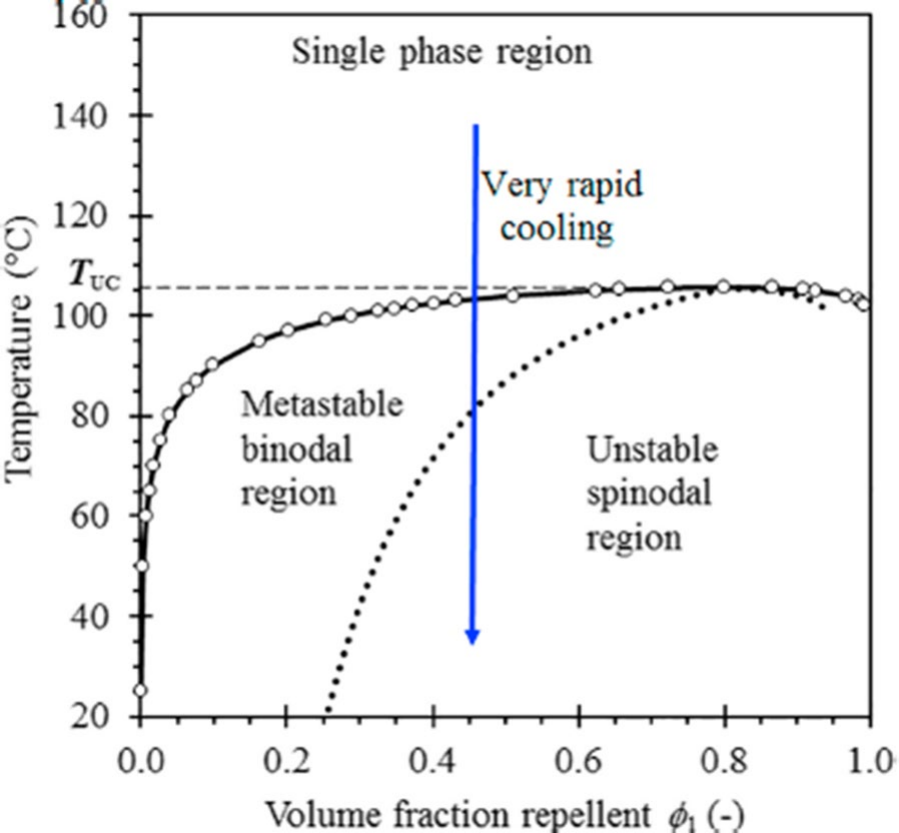
Phase diagram of the system linear low density polyethylene (LLDPE)/citronellal [14]. Republished with permission from Elsevier

The phase behaviour of a linear low density polyethylene (LLDPE)/citronellal system is illustrated in Fig. 17. The system shows an upper critical solution temperature (UCST) displaying the stable single-phase region together with the metastable and unstable regions. The phase diagram indicates that the probability of forming a microporous matrix is high when the polymer is in the minority phase. In polymer-repellent mixtures, the loci of the phase boundaries is described by the Flory-Huggins theory [124]. At temperatures above the UCST, the system is fully miscible for all compositions. Below this temperature, phase separation can occur at a temperature that depends on the concentration of the system components [125]. The composition of the two phases in equilibrium at any temperature are defined by the binodal line. In the metastable region indicated in the phase diagram, the phase separation will occur via a nucleation and growth mechanism [126]. This is the usual scenario for liquid-liquid phase separation [126]. If the polymer represents the minority phase, it may initially lead to the undesirable formation of separate polymer particles that are suspended in the continuous liquid repellent phase.

Inside the two-phase region there is another set of a phase envelope, the spinodal curves. In this region of the phase diagram, a homogeneous mixture is thermodynamically completely unstable. In contrast to the metastable bimodal region, the solution will spontaneously split into two phases through spinodal decomposition, into a polymer-rich phase and a solvent-rich phase. Phase separation through this mechanism leads to a finely dispersed microstructure through diffusion processes that amplify intrinsic thermodynamic spatial composition fluctuations. Ultimately this co-continuous structure may be fixed by either the subsequent crystallization of the polymer, or by vitrification of the polymer-rich phase. This means that the majority liquid phase is trapped inside a solid polymer-rich phase (which still may contain a minor amount of repellent) with a porous structure. In practice, such microporous microstructures are often achieved by rapid quenching of a homogeneous melt in a cold-water bath [14].

### Mechanism of release of repellent from microporous strand

**Fig. 18:**
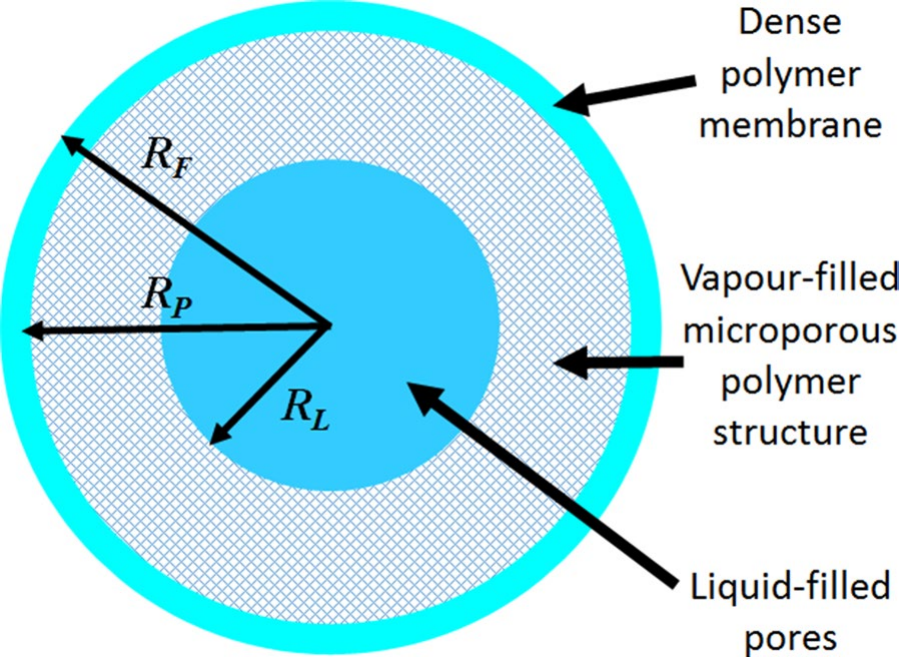
Schematic of a cylinder-shaped microporous membrane strand filled with repellent [14]. Republished with permission from Elsevier

In particular the preparation of phase-separated polymer/repellent systems, in which the repellent is entrapped in a porous polymer matrix and slowly released to the environment, is considered as an option to obtain a controlled drug release device/tool. Figure 18 shows the schematic of the cylinder-shaped microporous membrane-strand, which serves as a model of the repellent-release characteristics [14]. The cross-section is assumed to be circular, and the structure of the inner polymer section is assumed to be microporous. Conceptually it corresponds to an open-cell polymer foam, which is initially completely filled with the liquid repellent. As the repellent is gradually released into the atmosphere, it is assumed that the outer pores are progressively emptied and the lost liquid is replaced by air and repellent vapour. Further, it is assumed that the location of the liquid-vapour boundary is concentric with the outer wall [14]. In order for the active compound to be released from the polymer strand, a portion of the liquid evaporates and diffuses through the porous matrix towards the outer membrane. The matrix polymer forms both the microporous structure and the outer membrane. The permeability of the repellent through this membrane is defined by the product of its solubility in the membrane and the diffusion coefficient inside the membrane. The implication is that the active ingredient is also dissolved in the rest of the microporous polymer structure [14].

**Fig. 19:**
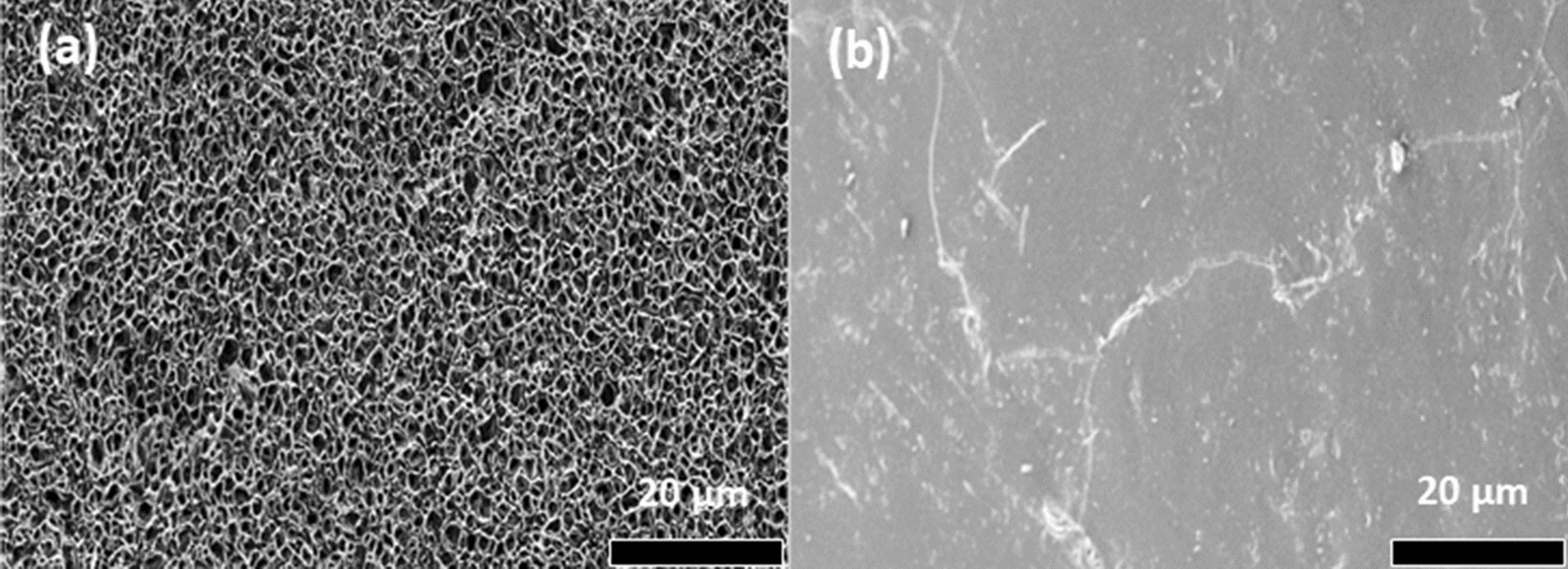
**a** SEM micrographs showing the internal structure of an extruded microporous LLDPE strap that contains 30 wt% DEET (an effective insect repellent), **b** the outer surface appearance of the skin [14]. Republished with permission from Elsevier

The geometric features of this model were analysed by SEM, presented in Fig. 19, and shows the microporous structure (Fig. 19a) of LLDPE initially containing 30 wt-% DEET covered by membrane (Fig. 19b) [14]. It is important to note that the microporous polymer structures act as reservoirs to trap large amounts of repellent. Most of the studies [13, 14, 117] used microporous polyolefins as carriers for repellents, because they are widely available and cost effective.

Although polyolefins have a detrimental impact on the environment in the event that used products are discarded or littered after use, polyolefins are the most extensively used group of thermoplastics due to acceptable strength, light weight, low cost, easy processability and good water barrier properties. This would make the total cost of the repellent-based product affordable. For this reason, the possibility was explored to develop repellent-based socks against outdoor mosquito bites [13]. Polyolefins are obtained through polymerization of olefins such as ethylene, propylene, butene, isoprene, and pentene, including their copolymers [127]. A fundamental common characteristic of all polyolefins is a nonpolar, nonporous, low-energy surface that is not receptive to inks and lacquers without special oxidative pre-treatment [128–130].

**Fig. 20:**
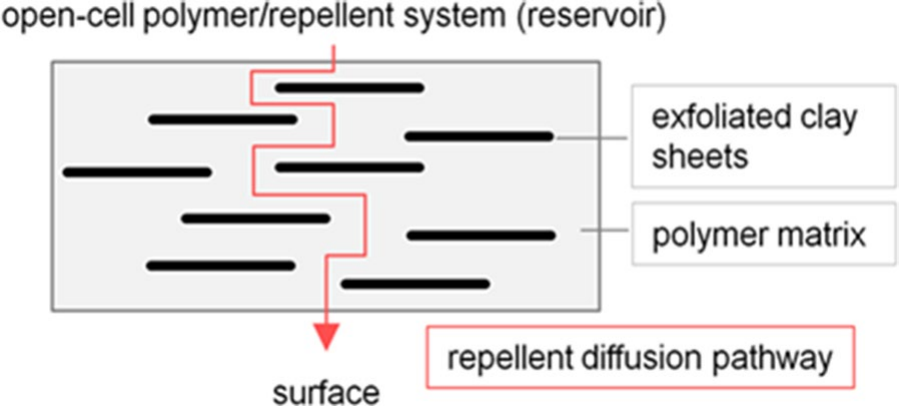
Sketch of well-dispersed exfoliated (isolated) and suitable oriented clay platelets in a polymer matrix, for control of the effective diffusion path for the repellent

Since the first production of polyolefins following the development of Ziegler-Natta catalysts, commercial exploitation has been very rapid because of their attractive characteristics. However, polyolefins are notch sensitive and brittle when exposed to low temperature and high rate of impact [130, 131]. In order to increase the application of polyolefins, fillers are incorporated into polyolefins to increase the stability, heat distortion, stiffness, strength and impact resistance without sacrificing their processability and barrier property [131–133]. In the case of malaria control, clay was added in some polymer-repellents formulations. Xu et al. [134] reported that intercalated or incomplete exfoliated structures and dispersed tactoids with several layers can effectively enhance the barrier properties of the polymer matrix. The aim of having exfoliated clay present is to control the release rate of the active ingredient, i.e., the volatile repellent, through the polymer membrane-like structure to the surfaces of the open-cell polymer-repellent system (Fig. 20). Therefore, when impermeable nanoparticles are added to a polymer, the permeating molecules are forced to wiggle around them in a random fashion, and consequently diffuse through a tortuous pathway [135].

A number of studies reported the development of intercalated or exfoliated nanocomposite structures with polyolefin/montmorillonite (MMT) [133, 136–142]. These studies revealed that the incorporation procedure into a polymer matrix is important in order to obtain complete nanoclay dispersion. The first work done by Mapossa et al. [14] reported about the influence of organoclay in the barrier property of polyolefin nanocomposite matrices against volatile mosquito repellents. Results demonstrated that the organoclay known as Dellite 43B added in the polymer lead to the expected lowering of film permeability as well as in reduction of release rate of repellents from microporous strands.

**Fig. 21:**
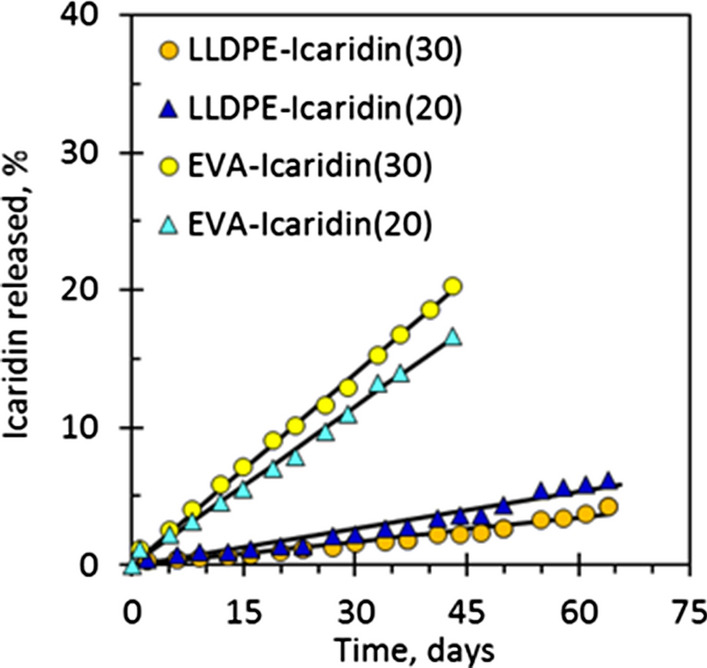
The constant release of repellents based on microporous poly(ethylene-co-vinyl acetate) (EVA) and linear low-density polyethylene (LLDPE) strands [14]. Republished with permission from Elsevier

For long-lasting protection against mosquitoes, the concentration of active ingredient in the repellent should have a constant rate of release during a sufficiently prolonged period. Work done by Mapossa et al. [14] showed that the mosquito repellent release from microporous straps can persist for several months at a constant rate. This is demonstrated by the constant slopes of the release curves in Fig. 21, suggesting that they can be developed into cost-effective long-life insect repellent systems. Furthermore, Brade and Davis [143] investigated the release of chemicals from a porous polymer. The authors used methyl nonyl ketone (KNK), dimethyl phthalate (DMP) and DEET as repellents. The porous polymer used was made from polypropylene. The results exhibited a slow release rate of DEET from porous polypropylene powder, being constant for 90 days.

A repellent dissolved in a polymer matrix may be released *via* a blooming mechanism. A disadvantage of this approach, however, would be an exponential decay of the release rate over time, initially being high but later, when still needed, being insufficient. Further disadvantages of this approach are a limited solubility of the repellent in the polymer device, and swelling of the polymer if the repellent is dissolved in the matrix. The latter means that the polymer will progressively shrink as the active substance is released, affecting the dimensional stability of the product. However, studies showed that the controlled-release technology based on polymer matrices are used largely due their low cost and versatility [144]. The mechanisms involved in slow release require polymers with a range of specific physicochemical properties [145] such as structure, chain length, molecular weight, and solubility [146].

Previous studies by [12–14, 123] show that microporous polymers have been used as carriers for the controlled release of volatile repellents. For example, a study conducted by Mapossa et al. [14] demonstrated that the microporous polymers namely poly(ethylene-co-vinyl acetate) (EVA) and linear low-density polyethylene (LLDPE) containing repellents DEET and Icaridin, provided a long protection time of up to 12 weeks against *An. arabiensis*. Chattopadhyay et al. [147] investigated the repellence activity of an essential oil (from the bark of cinnamon, leaves of lemongrass and leaves of eucalyptus)-based ethylcellulose and polyvinylpyrrolidone polymers patch against *Aedes albopictus*. The product, however, provided only up to three hours protection. In addition, Islam et al. [148] investigated the stability of ethyl-anthranilate-based polymer matrix for prophylaxis against vector-borne diseases.

The results showed that the obtained product was successful and the ethyl anthranilate-based product stayed stable for six months under the conditions studied without significant changes. These studies demonstrated the suitability of different ways of incorporating mosquito repellent into polymer matrices and proved their effectiveness against insects. The polyolefins used for designing different systems not only enhanced the physical-chemical stability but also the safety by entrapping the volatile compounds internally and releasing them at a desired controlled rate [148]. Additionally, previous studies about the efficacy of mosquito repellents, repellent-based controlled release systems and repellent-based products available on the market against mosquitoes are presented in Table 1.

From Table 1, it can be seen that some of the repellent-based products available on the market continue to have problems related to the short time of protection. The studies showed that the time of protection for topical formulations of natural and synthetic repellents against several mosquitoes range from one to 14 h. However, DEET-filled bicomponent fibres knitted into socks provided effectiveness against *An. arabiensis* for up to 20 weeks [13]. Furthermore, DEET remains the most studied, efficient and effective mosquito repellent. Due to some cases of toxicity related to the use of DEET reported in literature [149, 150], both IR3535 and Icaridin may be considered as alternative mosquito repellents. This is confirmed with the results obtained by Mapossa et al. [14] that demonstrated that polymer (LLDPE and EVA) strands with 20 % and 30 % of Icaridin provided a good repellency activity against *An. arabiensis* for up 12 weeks.

### Stability of the microporous polymers as carriers of insect repellent

A study conducted in our research group evaluated the thermal-oxidative stability of repellents at elevated temperatures using oven ageing [151]. The work aimed to determine whether the repellents could withstand short-time exposure to such high temperatures because during extrusion of the microporous strands, the mosquito repellents were exposed to typical polymer processing temperatures, i.e. *>*180 ^∘^C. Furthermore, the stability of relepellents during four months aged at 50 ^∘^C was investigated. Various repellents such as ethyl anthranilate, DEET, Icaridin, IR3535, dimethyl phthalate, decanoic acid and Citrodiol^®^. The results concluded that the repellents investigated were able to withstand typical polymer processing temperatures for short periods. That they also stayed essentially intact for several months at 50 ^∘^C suggesting that they may retain repellent activity for comparable lengths of time. In a study done by Sibanda et al. [13] showed that DEET is physically and chemically stable at melt fibre spinning conditions. Furthermore, a work done by Annandarajah et al. [152] demonstrated that pyrethrum and DEET were sufficiently thermally stable to be extrusion compounded with PLA.

## Factors affecting the efficacy of repellents

One of the key issues when trying to improve the effectiveness of an mosquito repellent is to control the volatility of the active ingredients. Optimum topical application is dependent on vapour phase repellence and prolonged duration [153]. In order to predict the effectiveness of repellents, it is imperative to understand the external factors that affect the repellents, particularly when they are applied on the skin, as well as products based on repellents existing on the market, for example creams, lotions, and sprays. The external factors include: abrasion, evaporation and temperature [153–156].

### Abrasion

This occurs through friction with clothing and other objects. This can also occur through other physical activities, which allows the repellent to be lost [153, 154, 157].

### Evaporation

**Fig. 22:**
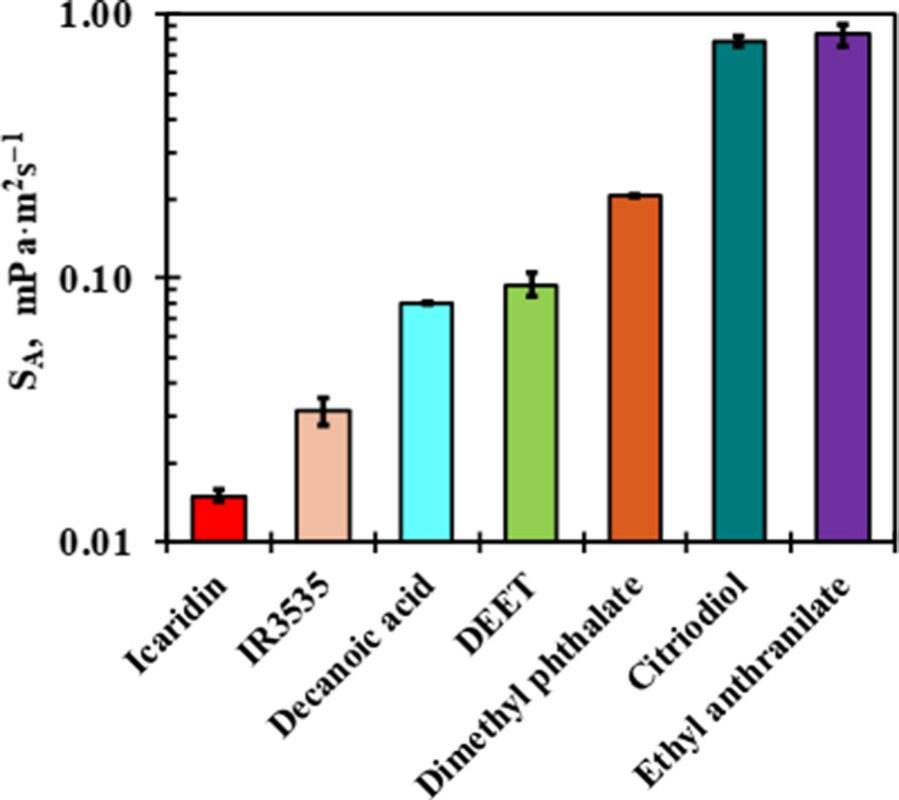
Evaporation rate of mosquitoes repellents measured at 50 °C [151]. Republished with permission from Wiley

The evaporation rate can be considered as one of the crucial physical properties of repellents which may affect their efficiency against mosquito bites, and this can be an indicator of the release rate of the repellent from polymer systems (i.e. microporous polymers matrices). This depends on the vapour pressure at ambient temperature and is associated to the boiling points of the repellents. Compounds that have a lower boiling point may allow better vapour repellence, but they may dispel faster. Compounds with higher boiling points have a low vapour pressure and would be ineffective in repelling at a pre-defined distance. This may allow mosquitoes to land but not bite. Generally, most repellents are effective up to a distance of about 4 cm of the skin [153, 154]. Mapossa et al. [151] evaluated the volatility of the different repellents using payne cups and thermogravimetric analysis. This study concluded that, if the repellent has a low volatility, it may provide effective protection. The repellents such as Icaridin and IR3535 were found as promising candidates for long time of protection against mosquito bites. Figure 22 shows the volatility of repellents conducted in convection oven at 50 °C. The repellents volatility was determined using the Eq. 1 that describes diffusion-controlled evaporation through a stagnant gas [158].

1 $$\frac{{d{m_A}}}{{dt}}\,\,\,\,=\,\,\,\left( {\frac{{{M_A}A\,}}{{zR\,T}}} \right)\,P_{A}^{{sat}}{D_A}$$

where ${{d{m_A}} {\left/ {\vphantom {{d{m_A}} {dt}}} \right. \kern-0pt} {dt}}$ represents TGA-measured rate of weight loss in (kg⋅s^−1^); $P_{A}^{{sat}}$ represents vapour pressure in (Pa) of sample at absolute temperature *T* in (K); *R* indicates the gas constant (8.3145 J⋅mol^−1^ K^−1^); *D*_A_ shows confficient of diffusion in (m^2^ s^−1^) of the repellent in air; *M*_A_ indicates the molar mass in (g⋅mol^−1^) of the repellent, *A* corresponds the cross-sectional surface area in (m^2^) of the cup, and *z* is the depth in (m) of the gas filled part of the sample cup.

Previous studies considered the link between the evaporation rate of repellents in relation to the protection period achieved against mosquitoes [154, 156, 159]. These results demonstrated that the time of protection was inversely proportional to the rate of evaporation of repellent.

### Temperature

This goes hand-in-hand with evaporation and concerns the effect of temperature on the evaporative loss of the repellent [153]. Khan et al. [160] investigated the influence of temperature on protection time of DEET and other repellents. They found that the protection time was halved with every 10 °C rise above ambient temperature, and that more repetitive application of the repellent was needed at temperatures over 26 °C. Further, a study conducted by Chung et al. [161] showed that the temperature affected the release rate of oil from microcapsules. The authors set the storage temperatures at 4, 25, and 60 °C and conducted the release property measurement of microcapsules. From the results they observed that the amount of released oil at 25 °C was slightly higher than that at 4 ^o^C. However, the release rate of oil was fast at 60 °C. At 60 °C, the mobility of molecules of the thyme oil became more active and thus volatile thyme oil rapidly diffused to the outside from the inside of microcapsules [161]. Similar behaviour was observed by Mapossa [162], when evaluating the release rate of DEET and Icaridin from LLDPE strands at 30 and 50 °C. As expected, the repellents DEET and Icaridin were released at a higher rate at a higher temperature of 50 than 30 °C [162]. The results obtained by the authors suggested that at an elevated temperature, this parameter affects evaporation of repellent as well as the protection duration of active ingredient. Additionally, other secondary factors such as wind velocity, loss from water wash-off and sweating also affected protection time.

## Mathematical modeling used for release rate of repellents

In order to provide predetermined release profiles, it is necessary to know the exact mass transfer mechanisms involved in the release of the repellents and to quantitatively predict the kinetics resulting from the release of the repellents. Therefore, it is needed to acquire a mathematical equation that describes the dependence of release as a function of time. The use of this tool is very useful to predict the release kinetics before the release systems are realized. This analytical solution conduces to many models that have been used to design a number of simple and complex drug (example, repellent) delivery systems or formulations, and to predict the overall release behaviour [163]. Mathematical models are an important tool to design pharmaceutical formulations, evaluate drug release processes in vitro, and in general, come up with the optimal design for new systems [164]. They allow the measurement of some important physical parameters (e.g., repellent diffusion coefficient) and resort to model fitting on experimental release data. The amount and type of active agent, polymer and adjuvants as well as the size and shape of the system designed to achieve a certain drug release profile can be predicted theoretically [163].

However, among the main-release kinetic models for release of repellents, the Higuchi, Korsmeyer–Peppas, Weibull and Mapossa models are presented. Table 3 presents a summary of the model release kinetics previously used to describe the release behaviour of active ingredient (repellent) most released through the polymeric membranes of microcapsules, nanoemulsion, and microporous polymer.

**Table 3:** Example of application of the mathematical models for release rate of repellents from different device systems and results descriptions obtained by authors in their studies

Equation models	Previous results description	References
Higuchi, Avrami’s or Weibull and Korsmeyer-Peppas equation models	The Higuchi model was employed to investigate the kinetic study of release of citronella oil from tamarind gum (TG) and carboxymethylated tamarind gum (CTG) microcapsules where the non-Fickian and Fickian diffusion mechanisms controlled the oil release. Furthermore, the use of Avrami’s model in those systems of release of citronella oil exhibited diffusion coefficient *n* < 1, indicating the Fickian diffusion mechanism that governed the systems. Finally, the Korsmeyer-Peppas model was also used to evaluate the release of oil from microcapsules. The prediction data from this model was fitted well with the experimental data. The parameter R^2^ was between 0.7642 to 0.9885, with a diffusion coefficient that demonstrated that the oil loaded into microcapsules was controlled by mechanism of Fickian and non - Fickian diffusion.	[29]
Higuchi zero-order, Higuchi and Korsmeyer-Peppas models	The mechanism of release of citronella oil from microcapsules was evaluated with three models known as Higuchi zero-order, Higuchi and Korsmeyer-Peppas. With Higuchi model it was possible to obtain a high parameter R^2^ of 0.9820 and the diffusion coefficient was close to 0.5, indicating that the oil loaded intro microcapsules device was governed by Fickian diffusion mechanism. While by use of Korsmeyer-Peppas model the parameter R^2^ was 0.9800 and the diffusion coefficient was close to 1, demonstrating that the release of oil from microcapsule was controlled by non-Fickian diffusion (anomalous diffusion) mechanism. Finally, the Higuchi zero-order model was a non-significant influence in release of citronella oil from the microcapsules.	[31]
Korsmeye-Peppas model	The release rate kinetic of neem oil from polymer microcapsules was investigated with the Ritger–Peppas model. The parameter R^2^ of neem oil into polymer microcapsules was linear. Further, the value of “*n*” obtained by use of model, showed that the release of neem oil from microcapsule is governed by Fickiam diffusion mechanism.	[28]
Higuchi, Korsmeyer-Peppas and Weibull models	Korsmeyer-Peppas model showed that the release of DEET from microcapsules was controlled by the Fickian diffusion mechanism. The prediction data obtained by the Peppa’s and Weibull models were in agreement to the experimental data of release of DEET from microcapsules. With Higuchi the constant R^2^ was lower than R^2^ obtained by other models.	[44]
Higuchi and Korsmeyer-Peppas models	The best correlation coefficient R^2^ equal to 0.9547 was obtained by use of the Korsmeyer-Peppas model for release of citronella oil loaded cotton microcapsules. The “*n*” value was equal to 0.5833, indicating that the system is controlled by anomalous non-Fickian diffusional mechanism. While for the release of oil of citronella loaded into polyester microcapsules, the best parameters R^2^ and “*n*” were 0.9477 and 0.3177, respectively. Therefore, the Fickian diffusion mechanism was observed.	[43]
Korsmeyer-Peppas model	The kinetic study of release of *Satureja hortensis* essential oil (SEO) from the alginate matrix was evaluated with the Korsmeyer-Peppas model. The predicted data obtained by the model was fitted with the experimental data with correlation coefficients R^2^ of three microparticles higher than 0.9. Furthermore, the parameter “*n”* was between 0.408 to 0.498, demonstrating that the mechanism of release rate of oil from microparticles was by Fickian diffusion.	[218]
Semi-empirical power law or Korsmeyer-Peppas model	The kinetic of release rate of the geraniol-to-zein ξ = 3 system, at different temperatures was determined. The results, in the range where 5 to 95 wt% of geraniol was evaporated, were fitted with the semi-empirical power law model. For a reservoir system with a spherical geometry with Fickian diffusion through the wall rate-limiting, the best fit value for the release exponent for the present data set was n = 0.80.	[219]
Mapossa model	This is a simple implicit mechanistic model used to predict the release rate of DEET and Icaridin from the microporous LLDPE strands that are covered by a skin like membrane that controls the release rate. In all cases, the model employed was a reasonable fit to the experimental data. This model assumes quasi-steady state diffusion and is based on the assumptions of a dimensionally stable and inert solid scaffold. This means that it will break down if the polymer absorbs and swells in the presence of the repellent. In this case, polyethylene is non-polar polymer, therefore, it was appropriate with this model.	[14]
Avrami’s equation	The Avrami’s equation was used to estimate the release rate of the limonene from nanomelusions systems. Results demonstrated that the release rate of repellent was controlled by the diffusion mechanism. The experimental and prediction data were fitted.	[179]
Avrami’s equation Higuchi model	To investigate the release of limonene oil from nanoemulsion, Avrami’s equation was employed. The results showed that the values of “*n”* for both homogenizations were almost in the same range of 0.6 to 1.0, suggesting that the release rate of limonene occurred through diffusion mechanism. The kinetic study of release rate of citronella oil from nanoemulsion was investigated by Higuchi’s model where the predicted data obtained by this equation was well fitted with the experimental data. Results from Higuchi’s equation, showed that the “*n*” value was equal to 0.5, suggesting that the release of citronella oil from nanoemulsion was controlled by diffusion mechanism.	[55] [56]
Higuchi and Korsmeyer-Peppas models	The Higuchi and Korsmeyer-Peppas models were employed to evaluate the citronella oil release kinetics from β-cyclodextrin. Among those models, the Korsmeyer-Peppas, R^2^ = 0.9877 and *n* = 0.6166 *±* 0.0275, when compared to the Higuchi model (R^2^ = 0.9751) showed better correlation and demonstrated a good fit between predicted data with the experimental data. The parameter “*n*” proved that the mechanism of release of oil from β-cyclodextrin was controlled by anomalous diffusion (0.5 < n < 1).	[88]
Korsmeyer-Peppas model	The Korsmeyer-Peppas model was employed to investigate the thyme oil release kinetic from β-cyclodextrin. The correlation coefficient for cotton fabrics treated with MCT- β -CD loaded with thyme oil was R^2^ = 0.9657 and parameter “n” was equal to 0.5444 demonstrating that the mechanism of release of oil was through anomalous diffusion mechanisms.	[188]
Korsmeyer-Peppas model	DEET was released slowly from the nanosphere systems. The study was governed by the diffusion mechanisms (Fickian diffusion and polymer relaxation). The device system maintained effective release rate of DEET, which has ensured performance activity times for more than nine hours. The prediction data obtained with the Korsmeyer-Peppas model was fitted well with experimental data.	[169]
Korsmeyer-Peppas model	The kinetic study of the amount of DEET released from polyurethane and polyurea microcapsules was evaluated using the Korsmeyer-Peppas model. The polyurethane microcapsules controlled the release rate of DEET well compared to the polyurea microcapsule. The mechanism of DEET release from polyurethane demonstrated “*n*” equal to 0.2120 while for DEET released from polyurea exhibited “n” equal to 0.2762. The results suggest that the non-Fickiam including diffusion as well as polymer relaxation mechanism was achieved.	[185]

### Higuchi model

**Fig. 23:**
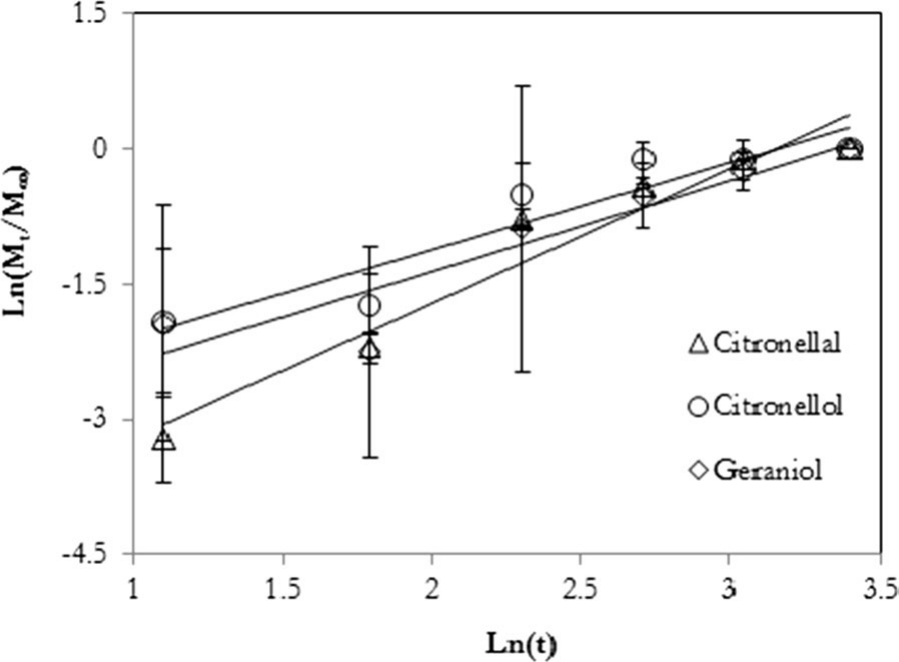
Experimental data of the repellent microcapsules release (symbols) and prediction data estimated using the Higuchi model [29]. Republished with permission from reference

The Higuchi equation is often used to analyse experimental release profiles, which may lead to erroneous conclusions about release mechanisms. The combination of different phenomena such as swelling, glassy/rubbery transitions, dissolution, and concentration-dependent diffusion may result in release data that display a square root dependence upon time [164]. Then, the release rate is given by Eq. 2.

2 $$\frac{{{{\rm{M}}_{\rm{t}}}}}{{{{\rm{M}}_\infty }}} = {\rm{K}}\sqrt {\rm{t}}$$

where, *M*_*t*_ and *M*_*∞*_ are the cumulative amounts of drug released at time *t* and infinite time, *K* is the Higuchi constant. Usually, this model describes repellent release as a diffusion process based on Fick’s law when the correlation coefficients (R^2^) is nearly 1.0. As an example, Fig. 23 represents the plot of slow release rate of citronella oil predicted using the Higuchi equation [29].

### Korsmeyer-Peppas model

The release of volatile actives from swellable polymers belongs to the category of diffusion problems known as moving-boundary or Stefan-Neumann problems [165]. Closed-form solutions are not generally available and it has become common practice to employ semi-empirical models to fit experimental data. The most widely applied models are due to Peppas and co-workers [165–168]. They realized that the behaviour of such systems are determined by two competing release mechanisms, i.e. Fickian diffusion and a Case II relaxational process. On this basis they developed two important semi-empirical release models on heuristic grounds [167]. They observed that, regardless of the geometric shape of the release device, the first 60 % of a release curve is adequately described by the so-called Korsmeyer-Peppas power law model represented by Eq. 3 [168].

3 $$\frac{{{{\rm{M}}_{\rm{t}}}}}{{{{\rm{M}}_\infty }}} = {\rm{K}}{{\rm{t}}^{\rm{n}}}$$

**Fig. 24:**
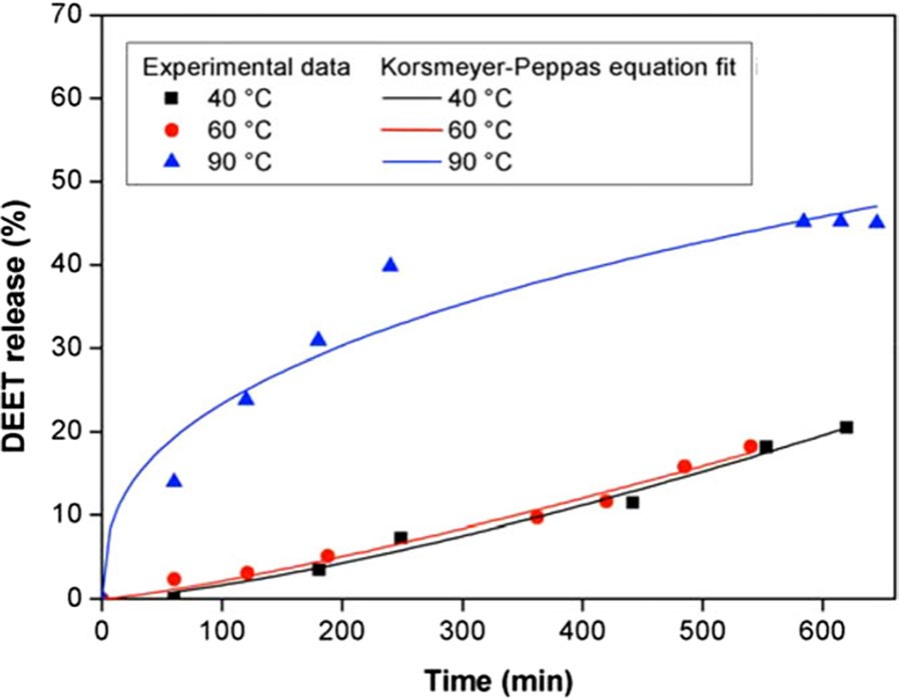
Experimental data of release rate of encapsulated DEET during three temperatures 40, 60, and 90 °C and prediction data predicted using Korsmeyer-Peppas model [169]. Republished with permission from Wiley

From Eq. 4, it can be represented as:

4 $$\ln \frac{{{{\rm{M}}_{\rm{t}}}}}{{{{\rm{M}}_\infty }}} = \ln {\rm{ k}} + {\rm{n}}\ln {\rm{t}}$$

where *M*_*t*_/*M*_*∞*_ is a fraction of core material released as the function of time *t, k* corresponds to the release rate constant and *n* is the diffusional exponent for active release. The diffusion mechanisms are represented as n = 0.5 indicating Fickian diffusion (Case I), n = 1.0 indicates relaxational transport (Case II), 0.5 < n < 1.0 corresponds non-Fickian diffusion (anomalous diffusion), and n > 1 corresponds Super Case II [169, 170]. The release rate of encapsulated repellent N,N-diethyl-3-methylbenzamide (DEET) is shown in Fig. 24. The Korsmeyer-Peppas model was used for data prediction.

### Weibull model

The Weibull model provides another globally valid expression for modelling release rates represented by Eq. 5:

5 $$X(t) = 1 - \exp [ - {(t/\tau )^n}]$$

**Fig. 25:**
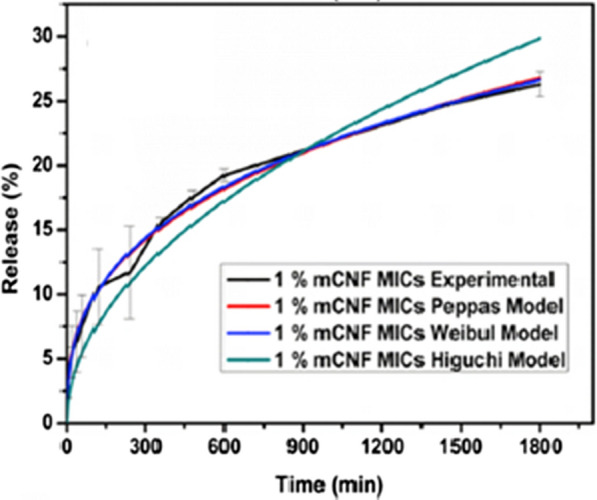
Experimental and predicted release rate profile of DEET from MICs [44]. Republished with permission from Elsevier

This expression is highly flexible and capable of correlating the behaviour of complex diffusion systems [171] including diffusion in fractal and disordered substrates [172]. The dimensionless exponent *n* is a shape parameter that determines the nature of the release curve. Interestingly, Weibull’s model also constitutes an extension of the Korsmeyer-Peppas model. This is because the exponent *n* is linearly associated to the exponent *n* of the power law derived from the analysis of the first 60 % of a curve of release [172]. Furthermore, the value that the exponent *n* indicates the transport mechanism of the active from the matrices of the polymer [172]. Values of *n* ≤ 0.75 represents Fickian diffusion mechanism [172]. Values in the range of 0.75 < *n* < 1 represents Fickian diffusion and Case II transport mechanisms. When *n* = 1, Weibull’s model reduces to classical first-order kinetics [173]. Complex release mechanisms are indicated when the *n* value exceeds unity [174, 175]. Figure 25 shows an experimental and predicted release rate profile of DEET from MICs by using Weibull’s model. The experimental and prection data are in close agreement, with R^2^ equal 0.9914.

### Mapossa model

**Fig. 26:**
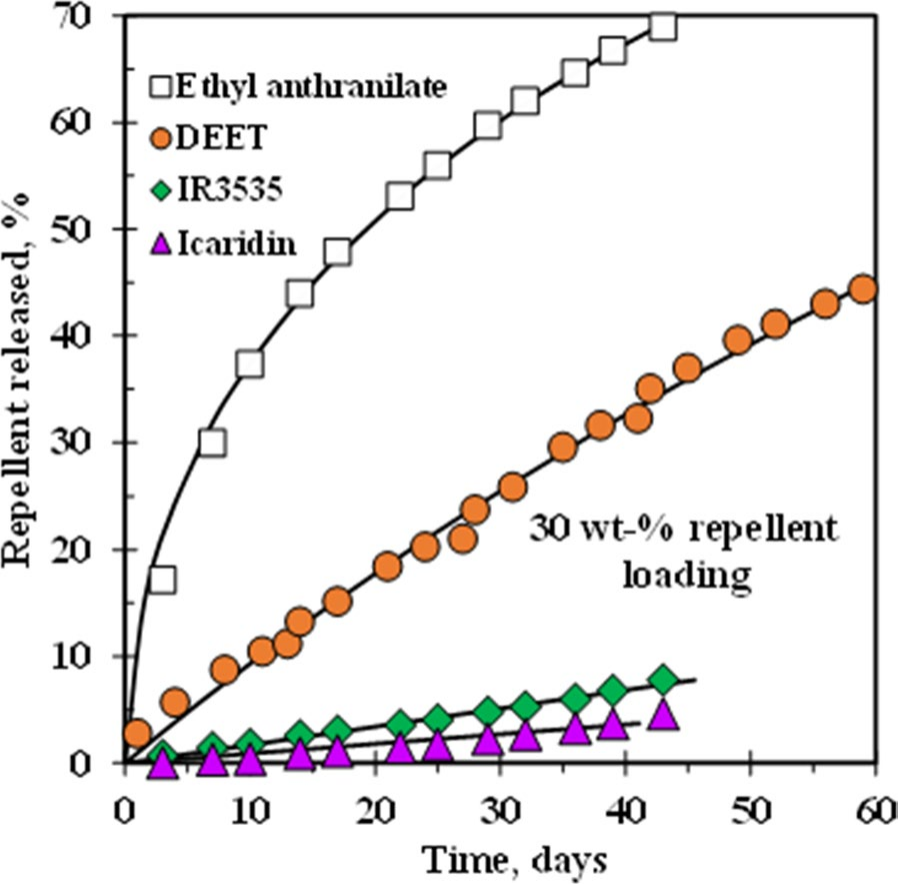
Experimental data of release rate of repellents from microporous LLDPE strands evaluated at 50 °C and estimation data predicted using Eq. (6) [151]. Republished with permission from Wiley

This model is defined by Eq. (non-polar, can be modelled with) that assumes quasi-steady state diffusion and is based on the assumptions of a dimensionally stable and inert solid scaffold. This means that it will break down if the polymer absorbs and swells in the presence of the repellent. In this case, polyethylene being non-polar, can be modelled with Eq. (6) [14, 176]:

6 $$t/\tau = \beta x + (1 - x)\ln (1 - x)$$

where *X* = *m*(*t*)/*m*(*t*→∞) represents the repellent mass (*m*) released after elapsed time (*t*) normalized with respect to the maximum that can be released; the characteristic time *τ* and the shape parameter *β* both depend on geometric features and the physical properties of the polymer device and mosquito repellent. Mapossa et al. [151] evaluated the release rate of different repellents, such as DEET, IR3535, Icaridin and Ethyl anthranilate from LLDPE strands covered by a thin mebrane barrier. The strands obtained from LLDPE approximated to this situation. The time dependent release from such strands followed the predictions of a simple implicit mechanistic model represented by Eq. 6. Therefore, the predicted data of release rate of repellents obtained by this equation was fitted well with the experimental data as is shown in Fig. 26.

Therefore, kinetic modelling of controlled release systems is necessary to predict the volatile compound release and protection time against insects. If the model is consistent, the behaviour of different combinations of active ingredients of compound and polymeric materials can be simulated at a reduced cost to achieve the desired performance [144]. Mathematical modelling of controlled release systems reduces the time and resources necessary for experimental work in product and process development (Additional file 1: Figure 1).

## Conclusions and recommendations for future work

Research on mosquito repellents is increasing every day due to the high demand for protection against mosquitoes-borne malaria. In the recent past, there has been extensive search for a safe, pleasant, and environmentally friendly product to mitigate or reduce transmission of diseases caused by mosquitoes. The most essential concern is to extend the time of protection of the repellents that are effective. The development of new tools as formulation-based mosquito repellents is an important strategy for achieving systems that are more effective and have fewer undesirable impacts. Repellent based on polymeric micro and nanocapsules, micro/solid lipid nanoparticles, nanoemulsions/microemulsions, liposomes, nanostructured micellar hydrogels and cyclodextrins provide slow release of mosquito repellent into the environment, improving the effectiveness of repellent for long period of time and reducing human exposure to the agent, for example, by permeation through the skin. Some of the studies suggested the possibility of developing long-life mosquito repellent-based products such as bracelets, socks, creams, roll-ons, and sprays that can be implemented in malaria-endemic regions outdoors. As a recommendation, more work should be done to understand their basic principles of formation and mechanism of release rate of repellent from the device’s systems. Additionally, more studies that emphasize the physical and chemical elements and basic entomological impact are also required. Finally, more extensive and rigorous entomological and epidemiological testing should be established on products-based repellents that are more refined before they could become commercially acceptable.

## Supplementary Information

**Additional file 1: Figure S1.** Chemical structure of: (a) DEET; (b) IR3535; (c) Icaridin and; (d) Ethyl anthranilate.

## Data Availability

All relevant data are provided in the manuscript or available from published materials as cited.
