# Supplementary material for: Mosquito‐repellent controlled‐release formulations for fighting infectious diseases
Source: Malar J. 2021 Mar 24;20:165. doi: 10.1186/s12936-021-03681-7 (PMC7988998; doi:10.1186/s12936-021-03681-7)
Supplement: Supplementary file 1 — Additional file 1: Figure S1. Chemical structure of: (a) DEET; (b) IR3535; (c) Icaridin and; (d) Ethyl anthranilate. [file 12936_2021_3681_MOESM1_ESM.docx]

Supplementary Material

**Mosquito-repellent controlled-release formulations for fighting infectious diseases**

António B. Mapossa^1,2*^, Walter W. Focke^1,2^, Robert K. Tewo^3^, René Androsch^4^, Taneshka Kruger^2^

^1^Institute of Applied Materials, Department of Chemical Engineering, University of Pretoria, Lynnwood Road, Pretoria, South Africa

^2^UP Institute for Sustainable Malaria Control & MRC Collaborating Centre for Malaria Research, School of Health Systems and Public Health, University of Pretoria, Private Bag X20, Hatfield 0028, Pretoria, South Africa

^3^Department of Chemical Engineering, Vaal University of Technology, Private Bag X021, Vanderbijlpark 1911, South Africa

^4^Interdisciplinary Center for Transfer-oriented Research in Natural Sciences, Martin Luther University Halle-Wittenberg, D-06099 Halle/Saale, Germany

*Corresponding author: [mapossabenjox@gmail.com](mailto:mapossabenjox@gmail.com)

## Vector control measures

Vector control measures play a significant role in the present global strategy of controlling the most important vector-borne diseases, mainly in the prevention of malaria [[1](#_ENREF_1)]. The most commonly used vector control interventions to prevent mosquito bites recommended by the WHO are indoor residual insecticide spraying and using long-lasting insecticide impregnated nets (LLINs). These methods are used in endemic regions such as sub-Saharan Africa [[2-4](#_ENREF_2)]. The methods were responsible for preventing two-thirds of malaria cases in Africa between 2000 and 2015 [[5](#_ENREF_5)]. Despite the showed success in reducing human-mosquito interactions, the methods are efficient simply against endophilic vectors [[6](#_ENREF_6)].

### *Indoor residual insecticide spraying*

IRS has long been recognised as the most commonly used method for malaria control. The use of dichloro-diphenyl-trichloroethane (DDT) against malaria has eliminated or significantly reduced the disease [[3](#_ENREF_3), [7](#_ENREF_7)]. Nowadays, IRS continues to be applied in many regions of the world, mainly Africa. In most cases, the services are provided by the public health system or by commercial companies [[3](#_ENREF_3)]. Indoor residual insecticide spraying with DDT has been the most effective chemical strategy against mosquitoes. However, DDT has its limitations which include the following: DDT does not last long, and its use has become uncertain. DDT is an organic pollutant and can persist for many years in the environment causing problems to public health [[8](#_ENREF_8)]. The possible effects of DDT exposure are low sperm counts, testicular anomalies, premature delivery of fetus and small gestational age for fetuses [[9](#_ENREF_9)]. The IRS method requires more complex and costly operational delivery systems than LLINs and claims of sustained high coverage often remain unproven [[10](#_ENREF_10)].

### *Long-lasting insecticide impregnated mosquito nets (LLINs)*

The use of mosquito nets as protection against harmful insects has been practiced since historical times [[11](#_ENREF_11)]. The WHO recommends coverage by LLINs for all people who live in regions at-risk for malaria. The most cost-effective way to achieve this is providing LLINs free of charge to ensure equal access of nets for all. Effective behaviour changes in communication strategies are also required to ensure that all people at risk of malaria sleep under a LLINs every night, and that the net is properly maintained. The LLINs interventions are used mostly in Africa. Unlike IRS they are low cost and easy to implement [[8](#_ENREF_8)]. In addition, these methods are very efficacious and effective [[11](#_ENREF_11)]. However, the principal limitation is that protection is only offered during sleeping time. It is also necessary to wash the nets from time to time, which gradually reduces their insecticidal property [[8](#_ENREF_8)]. In some countries in Africa, there is already evidence suggesting the emergence of vector resistance to insecticides, especially pyrethroids [[5](#_ENREF_5)].

The use of only indoor-based interventions has greatly reduced mosquito-borne diseases such as malaria. However, methods to control malaria in an outdoor environment need to be urgently developed as another alternative since people stay outdoors for lengthy periods of time during the day and early evening.

One of the limitations of the current vector control methods is insecticide resistance (especially pyrethroids). Insecticide resistance is the decrease of performance of insecticide against insects. This resistance can be observed when an insecticide repetitively fails to achieve the expected level of control when used according to the recommendations for the insect species. The growing development of insecticide resistance exhibited by various mosquito species poses a threat to malaria control programmes [[12](#_ENREF_12)].

Mosquitoes are developing resistance to groups of insecticides. In a study carried out on *An. arabiensis* from an area known as Gwave, a malaria endemic region in Zimbabwe, permethrin resistance in mosquito populations was discovered [[13](#_ENREF_13)]. In Côte d’Ivoire, resistance to permethrin, deltamethrin and λ-cyhalothrin was observed to be largely present in *Anopheles gambiae* [[12](#_ENREF_12)]. In Sudan, the WHO susceptibility assays with *Anopheles arabiensis* revealed resistance to DDT and pyrethroids [[14](#_ENREF_14)].

The growing trend of pyrethroid resistance constitutes a serious threat to malaria control programmes. Thus, the development of environmentally safe insect control methods and the rise of insecticide resistance have prompted research into repellents in recent years [[15-17](#_ENREF_15)]. Conducting research into repellents is challenging for several reasons, such as: (1) the different repellent phenomena are not well defined; (2) it is difficult to test and quantify repellence; (3) the physiological mechanisms are poorly known; and (4) the field efficacy appears to be highly variable [[17](#_ENREF_17)]. Previous studies proved and highlighted the use of repellents that can very well protect people by mosquito bites, thereby playing a significant role in reducing disease transmission [[15](#_ENREF_15), [16](#_ENREF_16), [18](#_ENREF_18), [19](#_ENREF_19)]. In addition, the use of devices that repel mosquitoes from a distance increased recently. These include impregnated plastic strips, coils and candles [[19](#_ENREF_19)]. [[20](#_ENREF_20)] obtained promising results incorporating repellents into polymer matrices to increase the time of repellence activity against mosquitoes.

Vaccines against yellow fever, Japanese encephalitis and tick-borne encephalitis are available in some places of the world [[21](#_ENREF_21), [22](#_ENREF_22)]. However, their limited access by the poor in endemic areas has prompted the development of alternative preventive measures to control the risk of the vectors [[15](#_ENREF_15)]. Therefore, in the absence of vaccines against malaria, one of the most effective and ancient prophylactic measures is the use of volatile mosquito repellents that may provide an additional line of defence against mosquito-borne diseases when used correctly and consistently [[15](#_ENREF_15), [23](#_ENREF_23)].

## Topical insect repellents

Insect repellents are known as volatile chemicals which, when applied on human skin, repel insects in the opposite direction from its source, thus discouraging contact and bites [[16](#_ENREF_16)]. It is also believed that most insect repellents act by producing a vapour barrier, which prevents contact of the insect with the human skin [[24](#_ENREF_24), [25](#_ENREF_25)]. Repellents are available on the market in many different chemical formulations such as aerosols, pump sprays, lotions, creams, suntan oils, powders, grease sticks and cloth impregnation laundry emulsions. Some of the factors that determine the suitability and the applicability of a repellent include: (i) type of repellent (e.g. active ingredient, formulations); environmental factors (temperature, humidity, wind), and (iii) inherent repellent properties (vapour pressure, boiling point, odour, solubility), as well as the mode of application and the attractiveness of individual people to insects [[26](#_ENREF_26)]. According to Islam et al. [[15](#_ENREF_15)] repellents tend to dispel rapidly and readily and may fail to protect against insects. For instance, repellents with low boiling points have a tendency to be less active as they vaporize too fast, providing a barrier only for a short period of time. Moreover, compounds with high boiling points have a propensity to have low efficacy as they do not vaporize readily and consequently do not produce sufficient vapours to form barriers [[27](#_ENREF_27)].

## *Characteristics of the ideal repellent*

The characteristics of the ideal insect repellent are the following: (a) good efficacy against a wide range of insects; (b) able to be used on the skin without side-effects; (c) neutral regarding damage clothing after application (i.e., staining, bleaching or weakening of fibres); (d) chemically stable; (e) economical availability and accessibiity for widespread use; (f) non-toxicity; (f) pleasant odour (no oily residues must be left on the skin that are difficult to remove by washing, wiping and sweating), and (g) inertness to most commonly used plastics [[16](#_ENREF_16), [28](#_ENREF_28)].

## *Insect repellent groups*

Mosquito repellents are divided in two categories: (1) synthetic repellents, which include DEET, IR3535^®^, Icaridin (trade name Saltidin^®^), and ethyl anthranilate or ethyl 2-aminobenzoate which nowadays dominate the market. Among these repellents, Icaridin and IR3535 are now considered candidates to substitte DEET in terms of repellence activity and (2) plant-derived repellents such as Lemon eucalyptus oil (Corymbia citriodora), Citronella oil (Cymbopogon nardus), Lipia javanica and Neem (Azadirachta indica).

### *Plant-derived repellents*

Essential oils (EOs) are complex mixtures of volatile organic compounds produced as secondary metabolites in plants. They are composed of hydrocarbons (terpenes and sesquiterpenes) and other oxygenated compounds (alcohols, esters, ethers, aldehydes, phenols, lactones) [[25](#_ENREF_25), [29](#_ENREF_29)].

For centuries plants have been used worldwide as medications to treat diseases. Relatively few plants gained significant attraction for use in controlling malaria-bearing mosquitoes and other arthropods. This could be due to the absence of scientific data rather than an absence of plant activity [[30](#_ENREF_30)]. Nowadays, the use of EOs from plants as insect repellents has a high consumer acceptance. This is due to the perception that natural repellents appear safer than synthetic ones and often are easier to acquire by people who live in the rural areas [[30](#_ENREF_30)]. Various EOs extracted from different plant families have been shown to have high repellence against arthropod species. For example, the monoterpenes limonene, citronellal, camphor, eugenol, terpinolene, and thymol are commonly described in the literature to have mosquito repellent activity [[25](#_ENREF_25), [31-33](#_ENREF_31)]. Among sesquiterpenes, ß-caryophyllene is most cited as a strong repellent against Aedes aegypti [[25](#_ENREF_25), [31](#_ENREF_31)]. The repellence activity of several essential oils seems to be associated with the presence of one or more volatile constituent substances (monoterpenes and sesquiterpenes) [[34](#_ENREF_34), [35](#_ENREF_35)]. According to researchers [[36-38](#_ENREF_36)] some repellents from plants are also effective against *Anopheles* mosquitoes. Yoon et al. [[39](#_ENREF_39)] reported the performance of essential oil extracted from *Cymbopogon genus* against *Aedes albopictus*, with its repellence activity tended to decrease over time, from 97.9 % at 0 h to 71.4 % at 1 h and 57.7 % at 2 h, as also demonstrated, the fennel oil effectiveness, from 88.6% at 0 h to 61.2% at 1 h and 47.4% at 2 h.

The research about repellents obtained from plants is increasing daily due to the consumer-demand for long-lasting mosquito-bite protection. The search is for a safe, pleasant and environmental-friendly product [[40](#_ENREF_40), [41](#_ENREF_41)]. Perhaps the most important consideration is improving the longevity of those repellents that are effective and most volatile such as citronella. Several studies looked at improving formulations of plant oils to increase their time of protection through the development of microencapusalions or nanoemulsions [[41](#_ENREF_41)]. Repellency activity of the limonene capsules-treated fabrics was evaluated against female mosquitoes *Culex pipiens* [[42](#_ENREF_42)]. Results demonstrated that although effectiveness of developed products decreased with washes, it displayed to be effective even after 20 washes.

*Eucalyptus oil (Corymbia citriodora)*

The active ingredient of eucalyptus oil includes 1,8-cineole, citronellal, citronellol, citronellyl acetate, eucamalol, limonene, linalool [[43](#_ENREF_43), [44](#_ENREF_44)]. Among them, citronellal is the most commonly used active ingredient against insects [[40](#_ENREF_40)]. Studies reported that eucalyptus oil based products can protect the human against insects bites up to 8 h according to their concentration [[44](#_ENREF_44)]. Further, Batish et al. [[44](#_ENREF_44)] also reported the effectiveness of 30 % eucalyptus oil can against mosquito bite for 2 h; however, the oil must have at least 70 % cineole content. The Center for Disease Control (CDC, USA) recommends the used of eucalyptus oil with active ingredient p-methane-3,8 diol (PMD) to protect against the West Nile virus which is a principal cause of mosquito-borne disease. This causes neurological disease. West Nile virus causes neurological disease or even death [[44](#_ENREF_44)]. It is common spread to people by the infected mosquito bite.

*Citronella oil (Cymbopogon nardus)*

It is one of the essential oils extracted from various species of *Cymbopogon*. Citronella oil is a common active ingredient in various perfumes and cosmetic products and is also well known for its use in outdoor candles, sprays, and lotions. Studies reportthat the oil of citronella is effective against insects in particular on mosquitoes when it is applied directly on the human skin [[45](#_ENREF_45)]. There are no adverse effects reported [[46](#_ENREF_46)]. A work conducted by Trongtokit et al. [[47](#_ENREF_47)] showed that 40 % citronella oil provided repellency for around 7 to 8 h. Brown, and Hebert [[27](#_ENREF_27)] reported that the oil of citronella can be effective even overnight. However, due to quick evaporation, it causes short time of protection against insects, requiring multiple applications. Thus, the system of devices for slow realease of citronella oil may help to improve the efficacy of citronella oil with time. One of the devices can be the nanoemulsion which is most used to reduce the evaporation of essentials oils and increase the time of repellent proctetion. Tavares et al. [[40](#_ENREF_40)] reported that nanoemulsions offer droplets that cover the surface of the skin more efficiently by improving repellent action.

*Lippia javanica*

It is commonly found in South Africa where its leaves are used as a decongestant for colds and coughs [[48](#_ENREF_48)]. The essential oils extracted from Lippia javanica show an excellent performance against mosquitoes. It is important to note that the Lippia javanica contains several terpenoids, the major component being 3-methyl-6-(1-methylethylidene)-cyclohex-2-en-1-one [[49](#_ENREF_49)]. Those components are active ingredients that give a good action to the repellent. Studies reported that oils of Lippia Javanica applied to the skin against *Aedes aegypti* may remain active during 2 h exhibiting 100 % of protection [[40](#_ENREF_40)]. In addition, an investigation conducted in Mpumalanga Province, South Africa by [Govere, Durrheim [50]](#_ENREF_50) showed that the oil of Lippia javanica was effective against *Anopheles arabiensis,* offering a protection of 76.7 % even after 4 h. At 5 h the Lippia javanica provided appreciable protection of 59.3 % against *Anopheles arabiensis*.

*Neem (Azadirachta indica)*

It is most used as traditional medicine in India. Its essential oils are used as insect repellent and demonstrate appreciable protection [[51](#_ENREF_51)]. Studies reported that after 2 h of application, formulation with 7.5 % Neem having botanical synergist extract was found to be effective with 90 % of repellency against mosquito *Aedes aegypt* [[52](#_ENREF_52)]. Dua et al. [[53](#_ENREF_53)] reported that the efficacy, environmental safety and public acceptability of neem and neem-based products for control of crop pests has led to its adoption into various mosquito control areas.

*Catnip (Nepeia cataria L.)*

It contains Nepetalactone, a terpene constituted of 2 isoprene units as an active ingredient. Catnip oil is used as an environmentally-safe repellent and showed a good performance against insects. The lotion product is applied directly on the skin which makes the body repel insects. Polsomboon et al. [[54](#_ENREF_54)] reported that Catnip oil at a 5 % concentration demonstrated to be effective against *Aedes vigilax, Culex annulirostris, and Culex quinquefasciatus.* Birkett et al. [[55](#_ENREF_55)] reported that the *Catnip* oils demonstrated to be effective for Anopheles gambiae comparable with the DEET. Study conducted by Polsomboon et al. [[54](#_ENREF_54)] eavaluated the efectivens of oil of Catnip for mosquitoes Aedes aegypti and Anopheles harrisoni. 5 % of Catnip oil exhibited 94 % knock down during 1h against that for Aedes aegypti while at 5% of oil of catnip demonstrated 55 % knock down of Anopheles harrisoni for 1 h.

### *Limitations of the use of plant-derived repellents*

Although repellents derived from plants are effective when freshly applied, most essential oils volatilize quickly. Hence, they tend to provide a shorter time of protection than synthetic repellents [[35](#_ENREF_35), [56](#_ENREF_56), [57](#_ENREF_57)]. This can be avoided by development of formulations able to keep the active ingredients onto the skin for long time. In addition, due low boiling points of plant-derived repellents it also limits their incorporation into most polymers because during compounding extrusion, large amounts of repellent can be lost by volatilization. Previous studies also showed that the plant-derived repellents (essential oils) present high thermal decomposition tendency, and poor physiochemical stability which make them unstable [[58](#_ENREF_58)]. Due to the disadvantages of plant-derived repellents mentioned above, market dominance of formulations based on longer-lasting synthetic repellents seems justified.

### *Synthetic repellents*

Before the use of synthetic repellents, primarily, the oil of citronella was the most extensively used compound and the standard against which others were tested [[59](#_ENREF_59), [60](#_ENREF_60)]. There were also three synthetic repellents, namely: (i) dimethyl phthalate (DMP) discovered in 1929; Indalone (butyl-3,3-dihydro-2,2-dimethyl-4-oxo-2H-pyran-6-carboxylate) patented in 1937, and (iii) ethyl hexanediol, also known as Rutgers 612 which was first used in 1939. These were the important repellents during World War II. After the war, three chemicals known as formulation 6-2-2 or M-250 (a combination of six parts DMP and two parts each Indalone and Rutgers 612) were later introduced for use by the military [[27](#_ENREF_27), [60](#_ENREF_60)].

*N,N-diethyl-3-methylbenzamide (DEET, formerly N,N-Diethyl-m-toluamide)*

First, this repellent was revealed by the US Department of Agriculture and patented by the US Army in 1946. This is the most effective and most widely used mosquito repellent because it is inexpensive and has showed good repellence activity against mosquitoes. It was approved for community utilization in 1959 and until now, it is considered a standard reference repellent of the WHO [[61](#_ENREF_61), [62](#_ENREF_62)]. Although DEET is considered very effective, its use in children has been limited because some medical cases have been reported. These include dermatitis, allergic reactions, neurological (seizures) and cardiovascular side-effects, as well as encephalopathy, especially when the repellent is used inappropriately [[62](#_ENREF_62), [63](#_ENREF_63)]. However, the causal role of DEET in these cases is debatable and taking into account the extensive use of DEET worldwide (around 200 million applications per year), the number of reported adverse events is low [[64](#_ENREF_64)].

Previous studies revealed most satisfactory performance of DEET against mosquitoes compared to other repellents such as IR3535, soybean oil, and citronellal [[65](#_ENREF_65)]. The product based on DEET has provided longer-lasting protection against mosquito when compared to other repellents. A work done by Frances et al. [[66](#_ENREF_66)] demonstrated that the application of DEET formulations against *Culex vishnui, Culex gelidus* and *Culex tritaeniorhynchus* mosquitoes provided 87 % protection for up to 5 hours, and with 50 % of DEET formulation 95 % protection was provided for 8 hours.

*Ethyl 3-[acetyl(butyl)amino] propanoate (IR3535)*

This repellent has a chemical structure like that of the amino acid alanine. In Europe, the IR3535 is available for more than 20 years. At a concentration of 20 %, IR3535 is effective against *Anopheles* and *Aedes* mosquitoes for a period of four to six hours [[67](#_ENREF_67)]. Previous studies carried out in Liberia showed that IR3535 can repel more than 92 % of biting *Anopheles gambiae* and *Anopheles funestus* for six hours [[68](#_ENREF_68)]. Also, other studies suggest that IR3535 is an effective repellent for *Anopheles, Aedes* and *Culex* mosquitoes [[69](#_ENREF_69)]. The time of protection of IR3535 can be similar to DEET, however, it necessitates often reapplication each 6 to 8 h [[70](#_ENREF_70)]. There are no recommendations for its use or avoidance in children or during pregnancy [[16](#_ENREF_16)]. Tavares et al. [[40](#_ENREF_40)] reported that the application of pure IR3535 directly on skin is not suggested, because it is oily and doesn’t offer an excellent sensation, and it also may permeate in the skin which this is not desired. However, the development of IR3535 based products is justified. It will be safe and effective to be used in communities against mosquitoes. Studies proved a good performance of IR3535 in laboratory and field tests.

*Icaridin (KBR3023: sec-butyl 2-(2-hydroxyethyl) piperidine-1) carboxylate*

This repellent (Icaridin), also known as Saltidin^®^, is available in various markets worldwide for use against many types of insect such as mosquitoes, black flies and ticks. Diaz [[16](#_ENREF_16)] reported that Icaridin was first discovered in Europe in the 1990s and released in the USA. Icaridin evaporates slowly from the skin, therefore, this may confer Icaridin long time of protection when compared to DEET [[40](#_ENREF_40)]. At a concentration of 20 %, Icaridin has an effectiveness against *Anopheles* and *Aides* mosquitoes for at least four to six hours [[67](#_ENREF_67)]. Most of the formulations available on the market generally contain 5 % to 30 % Icaridin, offering a long time of action against mosquitoes [[64](#_ENREF_64)]. Previous studies carried out on acute toxicity, irritant effect and skin penetration show KBR 3023 to be acceptable for human use [[69](#_ENREF_69)]. Tavares et al. [[40](#_ENREF_40)] reported that products based on Icaridin with 5 up to 10 % of ingredient active are recommended to be used for children at an age above 6 months, as substitute fot DEET. The physical properties of Icaridin show that it is a colourless, clear, viscous liquid that is stable in light and heat [[69](#_ENREF_69)].

*Ethyl anthranilate (EA)*

It is also known as ethyl 2-aminobenzoate. Nowadays, EA has attracted important attention in different groups of research. Despite few studies having been reported on its efficacy against mosquitoes, EA is a novel repellent considered an eco-friendly potential candidate to replace DEET [[59](#_ENREF_59), [71](#_ENREF_71), [72](#_ENREF_72)]. It is non-toxic, and the US FDA approved it as a volatile food additive. A work done by Islam et al. [[26](#_ENREF_26)] investigated the performance of ethyl anthranilate against several species of mosquitoes. The results showed that the ethyl anthranilate had an effectiveness against *Aedes aegypti, Anopheles stephensi and Culex quinquefasciatus* for at least two to four hours. In addition, Zhao et al. [[73](#_ENREF_73)] investigated the toxicity of ethyl anthranilate against *Aedes albopictus*. The study demonstrated that 0.1 % concentrations of EA solutions was highly toxic to *Aedes albopictus* larvae, and the mortality rate was >90 % after 4 h of application. The results obtained by Islam et al. [[59](#_ENREF_59)] suggest that the development of ethyl anthranilate-based formulations is safe and effective since there are no reports on the toxicity of this repellent. The chemical structures of DEET, IR3535, Icaridin and ethyl anthranilate are shown in Figure 1.

**Figure 1.** Chemical structure of: (a) DEET; (b) IR3535; (c) Icaridin and; (d) Ethyl anthranilate

**Spatial repellency**

Spatial repellents create a mosquito-free space, thereby preventing the contact between insects and humans. They can be used to protect more than one person at a time by dispersing active ingredients into the surrounding air that interferes with the mosquito’s ability to find a host [26, 74]. Repellent in space may interfere with host detection through excito-repellency, causing insects to fly in an undirected manner until they eventually move away from the source of repellent vapour [75]. Most spatial repellents are volatile pyrethroids, although some other active ingredients are available [76]. Achee et al. [77] reported that the behavior modification of probable vectors caused by spatial repellents has the potential to change the dynamics of pathogen transmission. The spatial repellency of Ethyl anthranilate (EA) was evaluated and compared with the commercial repellents [26]. The results demonstrated that in spatial repellency evaluation, EA was found extremely effective in repelling all the three mosquito species, hence displaying superior efficacy than many commercial repellents. As the anthranilate class of compounds contains a large diversity of natural chemicals, hence present many structural substitutes, thus could be exploited as good repellents [26]. From this argument we can assume that most of the natural repellents due their high volatility, they can be excellent candidate for spatial repellency against insects. In addition, the N,N-diethylphenyl acetamide (DEPA) was also investigated by Islam et al. [26], where also exhibited spatial repellency to some extent against mosquitoes.

Depending on efficacy of the active ingredient (AI) and application modality, this would result in a vector-free (or greatly reduced / suppressed) area. The exceptional benefit of spatial repellency (SR) is that the safe-zone can include specific areas both indoors and outside [77]. The volume of space that is protected or minimum protection range, will be dependent on the properties of the active ingredient, application platform and/or environmental conditions (e.g. air flow, temperature and humidity). Regardless of the particulars, the general concept of spatial repellency is clear: to discourage an arthropod from entering a space occupied by a potential human host thus reducing encounters between humans and vectors thereby eliminating or reducing the probability (risk) of pathogen transmission to either insect or human [76].

Study done by Achee et al. [77] about spatial repellents: from discovery and development to evidence-based validation, reported three summary points outlining role of spatial repellents and requirements for adoption in vector control such as: (a) The discovery, development and use of novel vector control tools will be required to achieve the goal of malaria elimination and eradication; (b) evidence exists of the benefits of sub-lethal approaches for interrupting human-vector contact but epidemiological data is insufficient to influence policy-makers to recommend spatial repellent tools for disease control confidently and (c) the adoption of a new paradigm shift in vector control to include behavior modification will require a new set of laboratory and field assay tools, standardized endpoints and analyses which must also be endorsed and adopted by leading global public health authorities.

Spatial repellents create a protective area within a given radius and can be used to protect more than one person at the same time. Dispersal of the active ingredient can be done in two ways, such as through heat, for example mosquito coils and electric emanators; or through evaporation, for example passive emanators made of paper or agarose gel [75]. Zhang et al. [78] reported that the most popular format of spatial repellent is the mosquito coil and it is estimated 45 to 50 billion mosquito coils are used annually by approximately two billion people worldwide, mainly in Southeast Asia. Mosquito coils are made from a mixture of inert ingredients, such as sawdust or coconut husks, and pigment. The coils burn at a low temperature dispersing the active ingredient, usually a volatile pyrethroid with a quick knock-down action (for example, pyrethrin, D-allethrin, transfluthrin, or metofluthrin). The smoke produced by the burning of mosquito coils can cause indoor air pollution [75].

Electric emanators consist of an electrical heating agent that vaporizes insecticide that has been impregnated into a pad or wick. These produce no smoke but require a source of electricity, which is not available in a large proportion of homes in malaria-endemic countries [75]. While the passive emanators do not require a source of heat or combustion. They have a large surface area which allows the passive dispersal of the volatile active ingredient into the air by evaporation. The chosen active ingredients are predominantly less polar compounds that are easily volatilized: examples include volatile pyrethroids such as metofluthrin and transfluthrin [75].

**References**

1. Zaim M, Guillet P. Alternative insecticides: an urgent need. Trends Parasitol. 2002;18:161-163.

2. Okumu FO, Moore SJ. Combining indoor residual spraying and insecticide-treated nets for malaria control in Africa: a review of possible outcomes and an outline of suggestions for the future. Malar J. 2011;10:208.

3. Pluess B, Tanser FC, Lengeler C, Sharp BL. Indoor residual spraying for preventing malaria. Cochrane database of systematic reviews. 2010. DOI:10.1002/14651858.CD006657.pub2.

4. Choi L, McIntyre S, Furnival-Adams J. Indoor residual spraying for preventing malaria (Protocol). Cochrane Database of Systematic Reviews, 2019. DOI:10.1002/14651858.CD013300.

5. Phillips MA, Goldberg DE. Toward a chemical vaccine for malaria. *Science*. 2018;*362*:1112-1113.

6. Reddy MR, Overgaard HJ, Abaga S, Reddy VP, Caccone A, Kiszewski AE, et al. Outdoor host seeking behaviour of Anopheles gambiae mosquitoes following initiation of malaria vector control on Bioko Island, Equatorial Guinea. Malar J. 2011;10:184.

7. Shiff C. Integrated approach to malaria control. Clinical microbiology reviews; 2002;15:278-293.

8. Sibanda MM. Polyolefin copolymers as controlled release devices for insecticides and repellents. https://repository.up.ac.za/handle/2263/56108 (2016). Accessed 23 Feb 2021.

9. Jaga K, Dharmani C. Global surveillance of DDT and DDE levels in human tissues. J Occup Med Environ Health. 2003;16:7-20.

10. Kleinschmidt I, Schwabe C, Shiva M, Segura JL, Sima V, Mabunda SJA, et al. Combining indoor residual spraying and insecticide-treated net interventions. Am J Trop Med Hyg. 2009;81:519-524.

11. Lengeler C. Insecticide‐treated bed nets and curtains for preventing malaria. The Cochrane Library, 2004.

12. Alou LPA, Koffi AA, Adja MA, Assi SB, Kouassi PK, N’Guessan R. Status of pyrethroid resistance in Anopheles gambiae ss M form prior to the scaling up of Long Lasting Insecticidal Nets (LLINs) in Adzope, Eastern Cote d’Ivoire. Parasites Vectors. 2012;5:1-6.

13. Munhenga G, Masendu HT, Brooke BD, Hunt RH, Koekemoer LK. Pyrethroid resistance in the major malaria vector Anopheles arabiensis from Gwave, a malaria-endemic area in Zimbabwe. *Malar J. 2008;7*:1-11.

14. Abdalla H, Wilding CS, Nardini L, Pignatelli P, Koekemoer LL, Ranson H, et al. Insecticide resistance in Anopheles arabiensis in Sudan: temporal trends and underlying mechanisms. Parasites vectors. 2014;7:1-9.

15. Islam J, Zaman K, Duarah S, Raju PS, Chattopadhyay P. Mosquito repellents: an insight into the chronological perspectives and novel discoveries. Acta Trop. 2017;167:216-30.

16. Diaz JH. Chemical and plant-based insect repellents: efficacy, safety, and toxicity. Wilderness Environ Med. 2016;27:153-163.

17. Deletre E, Schatz B, Bourguet D, Chandre F, Williams L, Ratnadass A, et al. Prospects for repellent in pest control: current developments and future challenges. Chemoecology. 2016;26:127-142.

18. Auysawasdi N, Chuntranuluck S, Phasomkusolsil S, Keeratinijakal V. Improving the effectiveness of three essential oils against Aedes aegypti (Linn.) and Anopheles dirus (Peyton and Harrison). Parasitol Res. 2016;115:99-106.

19. Alpern JD, Dunlop SJ, Dolan BJ, Stauffer WM, Boulware, DR. Personal protection measures against mosquitoes, ticks, and other arthropods.  Med Clin. 2016;100:303-316.

20. Mapossa AB, Sibanda MM, Sitoe A, Focke WW, Braack L, Ndonyane C, et al. Microporous polyolefin strands as controlled-release devices for mosquito repellents. Chem Eng J. 2019;*360*:435-444.

21. Ishikawa T, Yamanaka A, Konishi E. A review of successful flavivirus vaccines and the problems with those flaviviruses for which vaccines are not yet available. Vaccine. 2014;32:1326-1337.

22. Heinz FX, Stiasny K. Flaviviruses and flavivirus vaccines. Vaccine. 2012;30:4301-4306.

23. Leal WS. The enigmatic reception of DEET—the gold standard of insect repellents. Curr Opin Insect. 2014;6:93-98.

24. Nogueira BT, Perdiz SJ, Ricci JE, Regina EMC. Polymer-based drug delivery systems applied to insects repellents devices: A review. Curr Drug Deliv. 2016;13:221-235.

25. Nerio LS, Olivero-Verbel J, Stashenko E. Repellent activity of essential oils: a review. Bioresour Technol. 2010;101:372-378.

26. Islam J, Zaman K, Tyagi V, Duarah S, Dhiman S, Chattopadhyay P. Protection against mosquito vectors Aedes aegypti, Anopheles stephensi and Culex quinquefasciatus using a novel insect repellent, ethyl anthranilate. Acta Trop. 2017;174:56-63.

27. Brown M, Hebert AA. Insect repellents: an overview. J Am Acad Dermatol. 1997;36:243-249.

28. Katz TM, Miller JH, Hebert AA. Insect repellents: historical perspectives and new developments. J Am Acad Dermatol. 2008;58:865-871.

29. Toloza AC, Lucia A, Zerba E, Masuh H, Picollo MI. Interspecific hybridization of Eucalyptus as a potential tool to improve the bioactivity of essential oils against permethrin-resistant head lice from Argentina. Bioresour Technol. 2008;99:7341-7347.

30. Tisgratog R, Sanguanpong U, Grieco JP, Ngoen-Kluan R, Chareonviriyaphap T. Plants traditionally used as mosquito repellents and the implication for their use in vector control. Acta Trop. 2016;157:136-144.

31. Gillij Y, Gleiser R, Zygadlo J. Mosquito repellent activity of essential oils of aromatic plants growing in Argentina. Bioresour Technol. 2008;99:2507-2515.

32. Yang YC, Lee EH, Lee HS, Lee DK, AhnYJ. Repellency of aromatic medicinal plant extracts and a steam distillate to Aedes aegypti. J Am Mosq Control Assoc. 2004;20:146-149.

33. Jantan I, Zaki ZM. Development of environment-friendly insect repellents from the leaf oils of selected Malaysian plants. ASEAN Review of Biodiversity and Environmental Conservafion (ARBEC).1998;6:1-7.

34. Gonçalves J, Figueira J, Rodrigues F, Camara JS. Headspace solid‐phase microextraction combined with mass spectrometry as a powerful analytical tool for profiling the terpenoid metabolomic pattern of hop‐essential oil derived from Saaz variety. J Sep Sci. 2012;35:2282-2296.

35. Trongtokit Y, Rongsriyam Y, Komalamisra N, Apiwathnasorn C. Comparative repellency of 38 essential oils against mosquito bites. Phytotherapy Research: An International Journal Devoted to Pharmacological and Toxicological Evaluation of Natural Product Derivatives. 2005;19:303-309.

36. Odalo JO, Omolo MO, Malebo H, Angira J, Njeru PM, Ndiege IO, et al. Repellency of essential oils of some plants from the Kenyan coast against Anopheles gambiae. Acta trop. 2005;95:210-218.

37. Tawatsin A, Wratten SD, Scott RR, Thavara U, Techadamrongsin Y. Repellency of volatile oils from plants against three mosquito vectors. J Vector Ecol. 2001;26:76-82.

38. Trigg J. Evaluation of a eucalyptus-based repellent against Anopheles spp. in Tanzania. J Am Mosq Control Assoc. 1996;12:243-246.

39. Yoon JK, Kim KC, Cho Y, Gwon YD, Cho HS, Heo Y, et al. Comparison of repellency effect of mosquito repellents for DEET, citronella, and fennel oil. J Parasitol Res. 2015; doi.org/10.1155/2015/361021.

40. Tavares M, da Silva MRM, de Siqueira LBDO, Rodrigues RAS, Bodjolle-d'Almeida L, Dos Santos EP, et al. Trends in insect repellent formulations: A review. Int J Pharm. 2018;539:190-209.

41. Maia MF, Moore SJ. Plant-based insect repellents: a review of their efficacy, development and testing. Malar J. 2011;*10*:1-15.

42. Türkoğlu GC, Sarıışık AM, Erkan G, Yıkılmaz MS, Kontart O. Micro-and nano-encapsulation of limonene and permethrin for mosquito repellent finishing of cotton textiles. Iran Polym J. 2020;29:321-329.

43. Chagas ACS, Domingues LF, Fantatto RR, Giglioti R, Oliveira MC, Oliveira DH, et al. In vitro and in vivo acaricide action of juvenoid analogs produced from the chemical modification of Cymbopogon spp. and Corymbia citriodora essential oil on the cattle tick Rhipicephalus (Boophilus) microplus. Vet Parasitol. 2014;205:277-284.

44. Batish DR, Singh HP, Kohli RK, Kaur S. Eucalyptus essential oil as a natural pesticide. Forest Ecol Manag. 2008;256:2166-2174.

45. Khounvilay K, Estevinho BN, Sittikijyothin W. Citronella oil microencapsulated in carboxymethylated tamarind gum and its controlled release. Eng. J. 2019;23:217-227.

46. Hsu WS, Yen JH, Wang YS. Formulas of components of citronella oil against mosquitoes (Aedes aegypti). J Environ Sci Health B. 2013;48:1014-1019.

47. Trongtokit Y, Curtis CF, Rongsriyam Y. Efficacy of repellent products against caged and free flying Anopheles stephensi mosquitoes. Southeast Asian J Trop Med Public Health. 2005;36:1423.

48. Viljoen AM, Subramoney SV, Van Vuuren SF, Başer KHC, Demirci B. The composition, geographical variation and antimicrobial activity of Lippia javanica (Verbenaceae) leaf essential oils. J Ethnopharmacol. 2005;96:271-277.

49. Manenzhe NJ, Potgieter N, Van Ree T. Composition and antimicrobial activities of volatile components of Lippia javanica. Phytochemistry. 2004;65:2333-2336.

50. Govere J, Durrheim DN, Du Toit N, Hunt RH, Coetzee M. Local plants as repellents against Anopheles arabiensis, in Mpumalanga Province, South Africa. Cent Afr J Med 2000;46:213-216.

51. Patel SM, Venkata KCN, Bhattacharyya P, Sethi G, Bishayee A. Potential of neem (Azadirachta indica L.) for prevention and treatment of oncologic diseases. Semin Cancer Biol. 2016;40:100-115.

52. Kumari A, Siddhiqa N, Sharma R, Patanjali PK. Activity enhancement of Neem (Azadirachta indica) based repellent cream using biodegradable botanical synergist. Int J Pharm Sci Res. 2017;9:19.

53. Dua VK, Pandey AC, Raghavendra K, Gupta A, Sharma T, Dash AP. Larvicidal activity of neem oil (Azadirachta indica) formulation against mosquitoes. Malar J. 2009;8:1-6.

54. Polsomboon S, Grieco JP, Achee NL, Chauhan KR, Tanasinchayakul S, Pothikasikorn J, et al. Behavioral responses of catnip (Nepeta cataria) by two species of mosquitoes, Aedes aegypti and Anopheles harrisoni, in Thailand. J Am Mosq Control Assoc. 2008;24:513-519.

55. Birkett MA, Hassanali A, Hoglund S, Pettersson, Pickett JA. Repellent activity of catmint, Nepeta cataria, and iridoid nepetalactone isomers against Afro-tropical mosquitoes, ixodid ticks and red poultry mites. Phytochemistry. 2011;72:109-114.

56. Carroll SP, Loye J. PMD, a registered botanical mosquito repellent with deet-like efficacy.  J Am Mosq Control Assoc. 2006;22:507-514.

57. Barasa SS, Ndiege IO, Lwande W, Hassanali A. Repellent activities of stereoisomers of p-menthane-3, 8-diols against Anopheles gambiae (Diptera: Culicidae). J Med Entomol. 2002;39:736-741.

58. Patra A, Shah ARN. Current developments in (Malaria) mosquito protective methods: A review paper. Int J Mosq Res. 2019;6:38-45.

59. Islam J, Zaman K, Chakrabarti S, Bora NS, Pathak MP, Mandal S, et al. Exploration of ethyl anthranilate-loaded monolithic matrix-type prophylactic polymeric patch. J Food Drug Anal. 2017;25:968-975.

60. Bissinger BW, Roe RM. Tick repellents: past, present, and future. Pestic Biochem Physiol. 2010;96:63-79.

61. Lupi E, Hatz C, Schlagenhauf P. The efficacy of repellents against Aedes, Anopheles, Culex and Ixodes spp.–A literature review. Travel Med Infect Dis. 2013;11:374-411.

62. Fradin MS. Mosquitoes and mosquito repellents: a clinician's guide. Ann Intern Med. 1998;128:931-940.

63. Koren G, Matsui D, Bailey B. DEET-based insect repellents: safety implications for children and pregnant and lactating women. Can Med Assoc J. 2003;169:209-212.

64. Hasler T, Fehr J, Held U, Schlagenhauf P. Use of repellents by travellers: a randomised, quantitative analysis of applied dosage and an evaluation of knowledge, attitudes and practices (KAP). Travel Med Infect Dis. 2019;28:27-33.

65. Fradin MS, Day J.F. Comparative efficacy of insect repellents against mosquito bites. N Engl J Med. 2002;347:13-18.

66. Linthicum CPEK. Effectiveness Of Repellent Formulations Containing Deet Against Mosquitoes In Northeastern Thailand'. J Am Mosq Control Assoc.1996;12:331-333.

67. Sorge F, Imbert P, Laurent C, Minodier P, Banerjee A, Khelfaoui F, et al. Children arthropod bites protective measures: insecticides and repellents. Arch Pediatr. 2007;14:1442-1450.

68. Marchio F. Insect repellent 3535: a new alternative to DEET. SÖFW. Seifen, Öle, Fette, Wachse. 1996;122:478-485.

69. Barnard DR. Biological assay methods for mosquito repellents. J Am Mosq Control Assoc. 2005;21:12-16.

70. Khater HF, Selim AM, Abouelella GA, Abouelella NA, Murugan K, Vaz NP, et al. Commercial mosquito repellents and their safety concerns, in Malaria. In: Kasenga EH, editor. London, United kingdom; 2019.

71. Afify A, Horlacher B, Roller J, Galizia CG. Different repellents for Aedes aegypti against blood-feeding and oviposition. PLoS One. 2014;9:103765.

72. Kain P, Boyle SM, Tharadra SK, Guda T, Pham C, Dahanukar A, Ray A. Odour receptors and neurons for DEET and new insect repellents. Nature. 2013;502:507-512.

73. Zhao R, Wang R, Zheng L, Zhou Y, Wang L, Zhao F, et al. Toxicity and repellency of two anthranilates against Aedes albopictus Skuse (Diptera: Culicidae). *Acta trop*. 2019;*200*:105171.

74. Achee NL, Grieco JP, Vatandoost H, Seixas G, Pinto J, Ching-Ng L, et al. Alternative strategies for mosquito-borne arbovirus control. PLoS Negl Trop Dis. 2019;*13*:e0006822.

75. Maia MF, Kliner M, Richardson M, Lengeler C, Moore SJ. Mosquito repellents for malaria prevention. *Cochrane Database of Systematic Reviews*; 2018. doi.org/10.1002/14651858.CD011595.pub2.

76. Bibbs CS, Kaufman PE. Volatile pyrethroids as a potential mosquito abatement tool: a review of pyrethroid-containing spatial repellents. J Integr Pest Manag. 2017;8:1–10

77. Achee NL, Bangs MJ, Farlow R, Killeen GF, Lindsay S, Logan JG, et al. Spatial repellents: from discovery and development to evidence-based validation. Malar J. 2012;11:1-9.

78. Zhang L, Jiang Z, Tong J, Wang Z, Han Z, Zhang J. Using charcoal as base material reduces mosquito coil emissions of toxins. Indoor air. 2010;20:176-184.
